# Conditions of malaria transmission in Dakar from 2007 to 2010

**DOI:** 10.1186/1475-2875-10-312

**Published:** 2011-10-21

**Authors:** Libasse Gadiaga, Vanessa Machault, Frédéric Pagès, Abdoulaye Gaye, Fanny Jarjaval, Lydie Godefroy, Birane Cissé, Jean-Pierre Lacaux, Cheikh Sokhna, Jean-François Trape, Christophe Rogier

**Affiliations:** 1 Institut de Recherche pour le Développement, UMR 198; Unité Mixte de Recherche 6236 (URMITE), Route des Pères Maristes, BP 1386 Dakar, Sénégal; 2 Unité Mixte de Recherche 6236 (URMITE), Institut de Recherche Biomédicale des Armées, Allée du Médecin colonel Jamot, Parc du Pharo, BP 60109, 13262 Marseille cedex 07, France; 3 Centre National d'Etudes Spatiales - Service Applications et Valorisation - 18 avenue Edouard Belin, 31401 Toulouse Cedex 9, France; 4 Observatoire Midi-Pyrénées, Laboratoire d'Aérologie, Centre National de la Recherche Scientifique, Université Paul Sabatier, 14 avenue Edouard Belin, 31400 Toulouse, France; 5 Ecole Doctorale Eau, Qualité et Usages de l'Eau (EDEQUE), Université Cheikh Anta DIOP, Dakar, Sénégal; 6 Institut Pasteur de Madagascar, B.P. 1274, Ambatofotsikely, 101 Antananarivo, Madagascar

**Keywords:** *Anopheles*, Dakar, malaria, entomology, *Plasmodium *transmission, Human Biting Rate

## Abstract

**Background:**

Previous studies in Dakar have highlighted the spatial and temporal heterogeneity of *Anopheles gambiae s.l*. biting rates. In order to improve the knowledge of the determinants of malaria transmission in this city, the present study reports the results of an extensive entomological survey that was conducted in 45 areas in Dakar from 2007 to 2010.

**Methods:**

Water collections were monitored for the presence of anopheline larvae. Adult mosquitoes were sampled by human landing collection. *Plasmodium falciparum *circumsporozoïte (CSP) protein indexes were measured by ELISA (enzyme-linked immunosorbent assay), and the entomological inoculation rates were calculated.

**Results:**

The presence of anopheline larvae were recorded in 1,015 out of 2,683 observations made from 325 water collections. A water pH of equal to or above 8.0, a water temperature that was equal to or above 30°C, the absence of larvivorous fishes, the wet season, the presence of surface vegetation, the persistence of water and location in a slightly urbanised area were significantly associated with the presence of anopheline larvae and/or with a higher density of anopheline larvae. Most of the larval habitats were observed in public areas, *i.e*., freely accessible.

A total of 496,310 adult mosquitoes were caught during 3096 person-nights, and 44967 of these specimens were identified as *An.gambiae s.l*. The mean *An. gambiae s.l*. human-biting rate ranged from 0.1 to 248.9 bites per person per night during the rainy season. *Anopheles arabiensis *(93.14%), *Anopheles melas *(6.83%) and *An. gambiae s.s*. M form (0.03%) were the three members of the *An. gambiae *complex. Fifty-two *An. arabiensis *and two *An. melas *specimens were CSP-positive, and the annual CSP index was 0.64% in 2007, 0.09% in 2008-2009 and 0.12% in 2009-2010. In the studied areas, the average EIR ranged from 0 to 17.6 infected bites per person during the entire transmission season.

**Conclusion:**

The spatial and temporal heterogeneity of *An. gambiae s.l*. larval density, adult human-biting rate (HBR) and malaria transmission in Dakar has been confirmed, and the environmental factors associated with this heterogeneity have been identified. These results pave the way for the creation of malaria risk maps and for a focused anti-vectorial control strategy.

## Background

Urban malaria is considered to be an emerging problem in Africa because the populations of most large African cities have grown exponentially over the last 30 years [1]. Furthermore, it has been estimated that by 2030, 54% of the African population are expected to live in cities [2,3]. Many studies have reported evidence of malaria transmission in urban areas, and although levels are usually lower than in peri-urban and rural areas [2,4], urban malaria is considered to be an emerging health problem of major importance in Africa [1]. In African cities, transmission is spatially heterogeneous and occurs in areas where conditions are favourable for malaria vectors [5-8]. Indeed, in urban settings, malaria risk heterogeneity is recorded over small distances due to the degrees of diversity of types of urbanization, density of the human population, quality of water and waste management, vector control measures, household factors, access to health care and patterns of human migration, which could import parasites from rural areas [4,9,10]. The occurrence of malaria in African towns has also been linked to agricultural practices [11-14], to the distance from breeding sites [15-19] and to the vegetation cover [19].

To optimise the use of human and financial resources of the programmes for malaria control and because of the heterogeneity of malaria transmission in towns, it is appropriate to guide vector control interventions in only the areas of transmission. When drawn at appropriate scales, entomological maps of the spatial and temporal distribution of malaria vector larvae and adults could provide valuable information for targeted malaria control and the selective allocation of resources. Drawing such maps could theoretically be based on exhaustive entomological studies that cover the entire city over several years, but identification of the environmental factors that are associated with malaria transmission and the remotely sensed measurement of those factors could lead the way to an operational malaria risk mapping. Indeed, over the past several decades, remote sensing (RS) and geographic information systems (GIS) have become tools for evaluating the environmental, meteorological and climatic factors that influence the geographical and temporal risk distribution of malaria [20-25]. From 2005 to 2007 in Dakar, entomological data have been collected from several areas [6,8], and a portion of these data have allowed for a preliminary step towards malaria risk mapping, which is based on remotely sensed data, by setting up two risk maps for the years 1996 and 2007 [26]. For more accurate and precise malaria risk mapping, the objective of the present study was to investigate the environmental conditions associated with malaria transmission in-depth in 45 studied areas of Dakar and its suburbs from the years 2007 to 2010.

## Methods

### Study site

**Figure 1:**
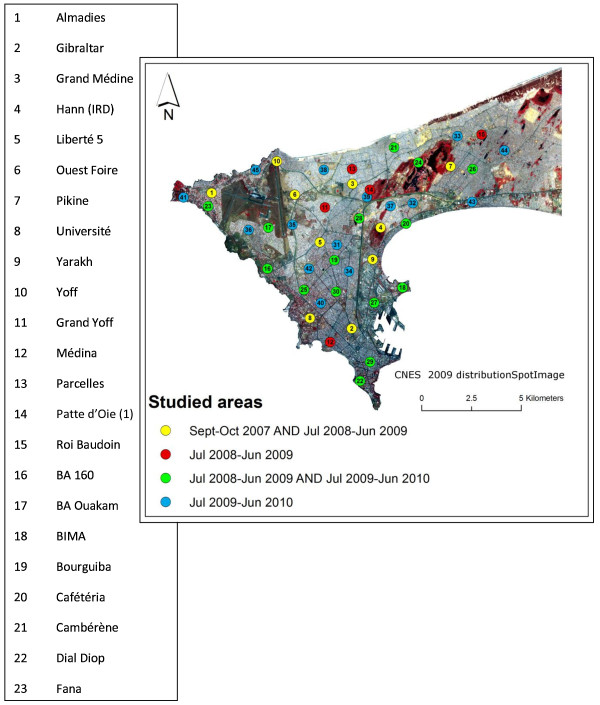
**Spatial distribution of 45 studied areas in Dakar and their period of study**.

Dakar (14°40'20" North, 17°25'22" West), the capital city of Senegal, is part of the Cap-Vert peninsula, which is located at the western-most point of Africa. The estimated population of Dakar was 1,030,594 inhabitants in 2005, which amounted to approximately 20% of the country's population, and the population density was 12,233 inhabitants per km^2^. The altitude of this area peaks at 104 m above sea level (Mamelles). The present study was conducted in 45 different areas of downtown Dakar and in Pikine, Thiaroye and Guediawaye, three of Dakar's satellite cities. The studied zones were chosen to cover as many diverse environments as possible in terms of type of urbanization, road network, vegetation and socio-economic level. Each zone was delimited on the ground to cover an area of approximately 200 × 200 m that depended on the technical and logistical limitations that were presented by the landscape. The studied zones are presented and listed in Figure 1, and each point is the centre of the area.

### Climate and study period

The Cap-Vert peninsula has a mild Sahelian climate. The hot and wet season occurs from June to November, the average temperature ranges from 24 to 30°C and the average rainfall amount is about 400 mm. The cool and dry season occurs from December to May, and the average temperature ranges from 19°C to 25°C. In 2007, 2008 and 2009, the annual rainfall was 178 mm, 510 mm and 565 mm, respectively. Field studies were undertaken in September and October of 2007 and from July of 2008 to June of 2010; therefore, three rainy seasons and two dry seasons were covered. In summary, the number of zones studied per year were as follows: 10 zones in September-October 2007 (see [8]), 30 zones in July 2008-June 2009 and 30 zones in July 2009-June 2010. Each zone was followed for one or two years, and the periods of study are presented in Figure 1. For those periods, daily precipitation values that were recorded at the international airport were available (data from the national weather agency). Two of the 45 zones (Bima and BA 160) were also studied in 2005 and 2006, but these studies followed a slightly different sampling methodology (see [6]).

### Mosquito larval sampling

#### Characterization of water collections

Each of the studied areas were visited every 10 days during the three rainy seasons and every month during the two dry seasons, in September-October 2007 and from June 2008 to April 2010 without any interruption. The physical, biological and chemical characteristics of all of the open water collections found in the studied areas were recorded. For visual classifications, consistency was maintained by having the same team perform the field work for the full duration of the project (two persons out of four rotated to perform this work). Subsequently, all of the collected information was transferred to a Microsoft Excel spreadsheet.

The type of habitat was categorised as ditches or puddles, swamp areas, marshes, ponds or lakes, "céanes" (non cemented wells used in market gardens), cemented wells or basins, waterproof containers, and canals. A water collection was considered to be temporary when it was found to be dry at least once during the follow-up; otherwise, it was recorded as permanent. The presence of larvivorous fishes, such as Guppies, Gambusia or Tilapia, was assessed visually. In 2007, the temperature of the water was measured using a mercury-in-glass thermometer that was immerged into the water for 60 seconds. From 2008 to 2010, a waterproof pH meter (PHSCAN 30, Hanna) provided the temperature, pH and conductivity of the water. Turbidity was estimated by using a graduated, transparent bottle that had black letters written on the bottom. The bottle was filled with water from the collection, and turbidity was evaluated by measuring the level that the water reached before the letters were no longer visible. Graduations ranged from 0 cm, at the top of the bottle, to 26 cm, at the bottom of the bottle; therefore, a higher value indicated greater turbidity. The proportion of the water surface that was covered by vegetation, *e.g.*, water lettuce, water lentils, grass or algae, was estimated visually, and the proportion of the shadow on the water at midday, which was dependent on the surroundings, was estimated visually. The salinity of the water was proportional to the conductivity.

#### Field larval sampling

All of the water collections in the 45 studied areas were examined for larvae. Larvae and pupae were sampled using a standard dipping method [27], which entailed performing a minimum of 20 dips for small collections and up to 100 dips for large water bodies. When anopheline specimens were found, the larval density was calculated as the number of larvae (all instars) and pupae, which were further emerged and identified at the laboratory, per dip and recorded for each water collection. The presence of *Culicinae *larvae was also recorded.

#### Larval mosquito molecular analysis

Random samples of the *Anopheles *specimen, which were found in the studied water bodies, were stored by date and breeding site in numbered vials that were filled with 70% alcohol. In the studied areas where adult *An. melas *had been caught, the species of the larvae specimens were identified by PCR (polymerase chain reaction). The PCR analyses were done on pools of specimens, and each pool corresponded to a sample that was collected at one breeding site on one date between September and October of 2007 and between July and September of 2008.

#### Larval sampling inside private properties during the 2009 rainy season

A transversal field study was undertaken in September of 2009 to investigate larval collections inside private properties. In each of the 45 studied zones, a random geographical sampling was performed using a GIS to choose the places that were to be visited. The sampling scheme included 30 private properties in each zone, which were numbered from 1 to 30, and the places were visited in the order of their numbers. When a private property was closed or if access was denied, the following property was visited, until a maximum of approximately 10 private properties were accessed. At every acceded property, the gardens, courtyards, balconies and flat roofs were searched for the presence of water bodies. A description of these water collections was recorded, and the presence of larvae was researched visually for small collections or by using a standard dipping method. These data were analysed separately from those that were collected outside private properties.

### Adult mosquitoes

#### Field adult mosquito sampling

Adult mosquito sampling was carried out by human landing catch, once every two weeks during September-October of 2007, during both extended 2008 and 2009 wet seasons (July to December), and once every month during both 2009 and 2010 dry seasons (January to June). For every studied zone, one catching point was located indoors and two were located outdoors except for Point E and Nord Foire. In the latter two areas, all three catching points were located outdoors, respectively from October of 2009 (and on the following 10 nights) and from September of 2009 (and on the following 10 nights), because it was not possible to find indoors catching points. Indoor captures were conducted by leaving a window or door slightly ajar. The three catching points were located around the centre of each studied area. Within each area, the distance between each of the three catching points was approximately 30 meters. Collectors gave prior informed consent and received yellow fever immunizations and anti-malarial chemoprophylaxis for the duration of the study and one month afterward. Two collectors were contracted to collect mosquitoes at each catching point from 7:00 p.m. to 7:00 a.m. or from 8:00 p.m. to 7:00 a.m., depending on the hours of dawn and dusk in Dakar, and each collector rested every two hours. Collectors were rotated among the catching points on different collection nights to minimise sampling bias. The mosquitoes were recorded by catching point, date and hour of capture, and they were then sorted by genera. The anopheline mosquitoes were identified morphologically following the keys of Gillies and Coetzee [28], and *Culicinae *were identified morphologically following the keys of Edwards [29]. All anopheline mosquitoes were individually stored in numbered vials with desiccant and preserved at -20°C until processing.

#### Molecular identification of *Anopheles gambiae s.l*

Depending on the number of *Anopheles *caught per studied area, all specimens, or a random sample of approximately 100 specimens, belonging to the *Anopheles gambiae *complex were selected from each study site for species identification by PCR [30]. All *Plasmodium falciparum *circumsporozoite protein (CSP)-positive anopheline mosquitoes were also tested.

#### Biting patterns and sporozoïtes rates

The human biting rate (HBR) was expressed as the average number of female *An. gambiae s.l*. bites per person per night for both outdoor and indoor catching points. For 2007, 2008 and 2009, the average values from September-October, which was the peak period of *Anopheles *densities (four collection nights in each studied area), provided a mean HBR for the rainy season (now named Rainy HBR). Values from November of 2008 to August of 2009 and from November of 2009 to June of 2010 were also averaged to calculate the mean HBR for the dry seasons (henceforth referred to as Dry HBR). The heads and thoraces of a quasi-exhaustive sampling of the adult *An. gambiae s.l *females that were caught on human bait were tested by enzyme-linked immunosorbent assay (ELISA) to detect the presence of the *P. falciparum *CSP [31].

The CSP index was calculated as the proportion of positive mosquitoes of the total number of ELISA-tested *An. gambiae s.l*. The CSP indexes were calculated for the 2007 rainy season (September-October), for July of 2008-June of 2009, and for July of 2009-June of 2010. The entomological inoculation rates (EIR) were calculated to be the products of the HBR and CSP indexes and provided the number of infected *Anopheles *bites per person per night. The EIR for the rainy seasons (henceforth referred to as Rainy EIR) was derived using the Rainy HBR and the CSP indexes. The Rainy EIR was calculated following two methods: by using the average CSP indexes for all areas together or by using the CSP indexes that were actually evaluated in each area and that took into account the number of infected mosquitoes that were caught in each area. The Rainy EIR was multiplied by 60 to provide the total number of infected *Anopheles *bites that could be received during the peak of transmission (September and October). For the studied area that was followed during more than one year, the CSP indexes were compared.

### Statistical analysis

The statistical analyses for the larval study aimed to identify the following: the determinants of the presence or absence of *Anopheles *larvae in the water collections, andthe factors associated with the density of *Anopheles *larvae at the breeding sites. Continuous independent variables were dichotomised at the mean or median, and the statistical unit was defined as the measurement of variables for a given water collection at one date. In longitudinal studies, some correlation may exist between observations made for the same water collection; therefore, to take this possible interdependence of observations into account, random effect models were used. The presence or absence of larvae was analysed using a logistic regression model, and the larval density was analysed using a negative binomial regression model with the number of larvae per 100 dips as the dependant variable. The variables associated with the presence or the density of the larvae with a p-value < 0.25 in univariate analysis were retained for multivariate analysis, and a backward, stepwise selection procedure was applied in the final model to keep variables with a p-value < 0.05 in the analysis. Proportions were compared using a chi-squared test or Fisher exact test, as appropriate, and all analyses were performed using STATA 9.0 software (Stata-Corp LP). Some of the 2007 results have been presented in [8], but all data from 2007 through 2010 were taken into account in the analysis to increase statistical power.

#### Correlation between larvae and adults densities

The adult density fraction was estimated for each of the studied areas by calculating the total number of *Anopheles *that were caught during one decade, and these numbers were divided by the total number of *Anopheles *that were caught during the entire studied year. The larval density fraction at each studied area was estimated as follows: the product of the larval density was multiplied by the water surface for each breeding site for a decade, the sum of these products for all of the water collections was calculated for the entire studied area, the resulting number was then divided by the yearly value, and correlations between the larval density fraction and adult density fraction were graphically examined.

## Results

### Mosquito larval sampling

#### Characterization of water collections

A total of 325 water collections were examined. Depending on the persistence of the water bodies and on their characteristics of draining, emptying and drying out, each water body was investigated between one to 37 times. The types of open water collections included the following: 203 ditches or puddles; 24 swamp areas, marshes, ponds or lakes; 70 "céanes", cemented wells or basins; 11 large artificial holes (type underpinning); nine small, waterproof containers (tyres or buckets); and eight canals. Among them, 261 (80%) were temporary and 64 (20%) were permanent. The description of the quantitative physical, biological and chemical parameters that were recorded for the water collections are presented in Additional file 1.

#### Field larval sampling

A total of 228 of the 325 (70%) water bodies were found to be breeding sites for *Anopheles *at least once during the follow-up period, and 57 were breeding sites at every date when water was present. Of the temporary collections, 191 (73%) were habitats for anopheline larvae at least once during the follow-up, while for the permanent collections, 37 (58%) were *Anopheles *breeding sites at least once. Several of the studied areas did not harbour any breeding site during the full duration of their follow-up (Grand Yoff, HLM, Nord Foire and Point E). In breeding habitats, the density of *Anopheles *larvae and pupae ranged from 0.01 per dip (in large permanent collections in Golf and Hann IRD), to 42 per dip (in a concrete basin of an abandoned market-garden in Yarakh in 2008).

#### Larval mosquito molecular analysis

In 2007, PCR amplification undertaken on *Anopheles *larvae highlighted the presence of *An. arabiensis *only (no *An. melas*) among *An. gambiae s.l*. larvae [8]. During the 2008 rainy season, a total of 164 pools of larvae were tested for species identification by PCR amplification, and 147 pools were found to be positive for *An. Arabiensis *only, 15 pools were positive for both *An. arabiensis *and *An. melas*, and two pools were positive for *An. melas *only. All of the pools that contained *An. melas *were collected from three areas (Cafetériat, Pikine or Zone A), and those samples were taken from a large marshy area (Niaye), artificial basins or puddles. Due to laboratory constraints, no pools from Bima, Cambérène, Golf, or Potou where tested. In the breeding sites containing *An. melas*, the conductivity of the water was found to be 5.03 (range: 0.3 to > 20; 95% CI: 1.43 - 8.62), while this value was found to be 1.75 (range: 0.06 to 12.03; 95% CI: 1.40 - 2.09) in the breeding sites that did not contain *An. melas*. This difference was significant (Student t-test p < 0.0001).

#### Longitudinal survey of breeding sites

A total of 2,903 observations were recorded in the 325 sampled water collections in October-September of 2007 and between July of 2008 and April of 2010. The presence or absence of *Anopheles *larvae was known for 2,683 of those observations (1015 observations, 38%, were positive for larvae), and the *Anopheles *larval density was known for 1008 of the observations. The results of the univariate and multivariate analyses of the presence of *Anopheles *larvae and of the *Anopheles *larval density are presented in Additional file 2 and Table 1. A water pH equal to or above 8.0, the absence of larvivorous fishes, the wet season, the presence of surface vegetation, the persistence of water collection and a location in a lowly urbanised area were significantly associated with the presence of anopheline larvae in open water collections. Among the water collections that contained anopheline larvae, a water temperature equal to or above 30°C, a water pH equal to or above 8.0, the absence of larvivorous fishes and the wet season were significantly associated with a higher density of anopheline larvae.

**Table 1:** Factors associated with the presence of *Anopheles *larvae and the *Anopheles *larval densities in the open water collections recorded in 45 studied areas in Dakar in October-September 2007 and between July 2008 and April 2010.

	Presence of *Anopheles *larvae			*Anopheles *larval density		
	**OR**	**95% CI**	**p-value**	**IRR**	**95% CI**	**p-value**

	2 273 observations		< 0.0001	852 observations		< 0.0001

Water temperature						

< 30°C				1		

> = 30°C				1.15	1.02 - 1.30	0.026

pH						

< 8	1			1		

> = 8	1.79	1.35 - 2.37	< 0.0001	1.31	1.15 - 1.49	< 0.0001

Presence of larvivorous fishes						

No	1			1		

Yes	0.52	0.34 - 0.83	0.006	0.70	0.55 - 0.89	0.003

Season						

Dry (Nov to Jun)	1			1		

Wet (Jul to Oct)	2.94	2.20 - 3.92	< 0.0001	1.35	1.15 - 1.59	< 0.0001

Surface vegetation (%)						

< 20%	1					

> = 20%	2.34	1.67 - 3.29	< 0.0001			

Persistence of water collection						

Permanent	1					

Temporary	7.21	3.83-13.58	< 0.0001			

Water collection located in highly urbanized area						

No	1					

Yes	0.05	0.02 - 0.19	< 0.0001			

Logistic regression and binomial negative regression with water collection random effect. Multivariate analysis.

#### Larval sampling inside private properties during the 2009 rainy season

Between five to 12 private properties or buildings that were not usually accessible for monitoring water collections were visited during September of 2009 in each of the 45 studied areas, totalling 355 private properties. In 153 of the private properties, no water collection or water container was found, but in the remaining 202 private properties, water was found in small containers (e.g., buckets, bowls, saucepans, and cans), in pools or basins, or in puddles that were located in gardens or on flat roofs or balconies. *Culicidae *larvae were found in 80 (23%) of the private properties, which were located in 27 of the study areas, and *Anopheles *larvae were found in 11 (3%) of the 355 private properties, which were located in 8 study areas (Cafétéria, BA Ouakam, Zone A, Dial Diop, Liberté 6 Extension, Thiaroye Mairie, Point E and Karack). Those breeding sites included the following: puddles in courtyards or gardens of houses or schools, water tanks, large cans, abandoned large containers, basins or swimming pools. No *Anopheles *larvae were found in small water containers.

### Adult mosquitoes

#### Field adult mosquito sampling

**Figure 2:**
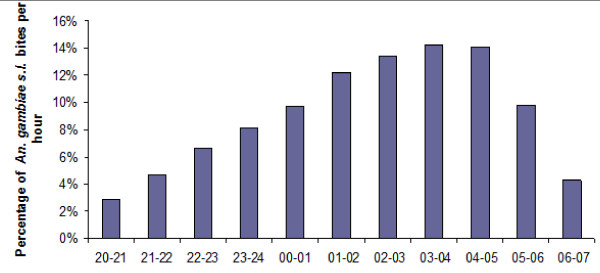
**Hourly distribution of *An. gambiae s.l*. bites in 45 studied areas in Dakar, from 2007 to 2010**.

A total of 496,310 human-landing mosquitoes were caught between the years 2007 and 2010, and this sampling occurred during 3096 person-nights (1,012 indoors and 2,084 outdoors) (Table 2). Among the 44,967 *An. gambiae s.l*. that were collected, 32,403 (72%) were caught outdoors, and 12,564 (28%) were caught indoors (Table 3). Cumulatively for the 45 studied areas, the peak biting time was between 1:00 a.m. and 5:00 a.m. (Figure 2), and 77.4% of the bites occurred after midnight.

**Table 2:** Distribution by genus and species of adult mosquitoes collected on humans in 45 studied areas in Dakar in Sept-Oct 2007, Jul 2008-Jun 2009 and Jul 2009-Jun 2010.

	Sept-Oct 2007 (10 areas, 120 person-nights of capture)		Jul 2008-Jun 2009 (30 areas, 1404 person-nights of capture)		Jul 2009-Jun 2010 (30 areas, 1572 person-nights of capture)		Total	
	**Nb**	**% of total population**	**Nb**	**% of total population**	**Nb**	**% of total population**	**Nb**	**% of total population**

*Anopheles gambiae s.l*.	1101	5.66%	20773	11.42%	23093	7.83%	44967	9.06%

*Anopheles pharoensis*	10	0.05%	298	0.16%	95	0.03%	403	0.08%

*Anopheles ziemani*			33	0.02%	93	0.03%	126	0.03%

*Culex quinquefasciatus*	14428	74.18%	136001	74.78%	257947	87.44%	408376	82.28%

*Culex tritaeniorynchus*	3010	15.47%	19475	10.71%	10 665	3.62%	33150	6.68%

*Aedes aegypti*	821	4.22%	4289	2.36%	1 839	0.62%	6949	1.40%

*Aedes metallicus*	1	0.01%	23	0.01%			24	0.00%

*Aedes vittatus*					8	0.00%	8	0.00%

*Aedes sp*.			11	0.01%	1	0.00%	12	0.00%

*Mansonia sp*.	80	0.41%	964	0.53%	1251	0.42%	2295	0.46%

Total	19451		181867		294992		496310	

**Table 3:** Outdoors and indoors distribution of adult *An. gambiae s.l*. collected on human bait in 45 studied areas in Dakar in Sept-Oct 2007, Jul 2008-Jun 2009 and Jul 2009-Jun 2010.

	Sept-Oct 2007 (10 areas)		Jul 2008-Jun 2009 (30 areas)		Jul 2009-Jun 2010 (30 ares)		Total	
	**Number of person-nights**	**Number of *An. gambiae s.l*.**	**Number of person-nights**	**Number of *An. gambiae s.l*.**	**Number of person-nights**	**Number of *An. gambiae s.l*.**	**Number of person-nights**	**Number of *An. gambiae s.l*.**

Outdoors	80	870	936	14443	1068	17090	2084	32403

% outdoors	67%	79%	67%	70%	68%	74%	67%	72%

								

Indoors	40	231	468	6330	504	6003	1012	12564

% indoors	33%	21%	33%	30%	32%	26%	33%	28%

								

Total	120	1101	1404	20773	1572	23093	3096	44967

#### Molecular identification of *An. gambiae s.l*

Of the 3,775 specimens of *An. gambiae s.l*. that were successfully tested by PCR, 3,516 were identified as *An. arabiensis *(93.14%), 258 were identified as *An. melas *(6.83%), and one was identified as *An. gambiae s.s*. M form (0.03%) (Additional file 3). The highest densities of *An. melas *were found in Hann (IRD) and Golf during the 2008-2009 and 2009-2010 seasons, respectively, where this species accounted for more than 40% of the *An. gambiae s.l*. population.

#### Biting patterns and sporozoïte rates

**Figure 3:**
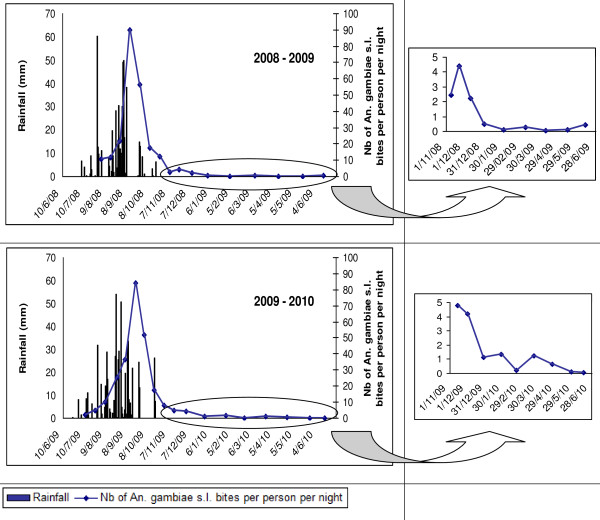
***Anopheles gambiae s.l*. HBR and rainfall events in 10 studied areas in September-October 2007, 30 studied areas in July 2008 - June 2009 and 30 studied areas in July 2009 - June 2010**.

**Figure 4:**
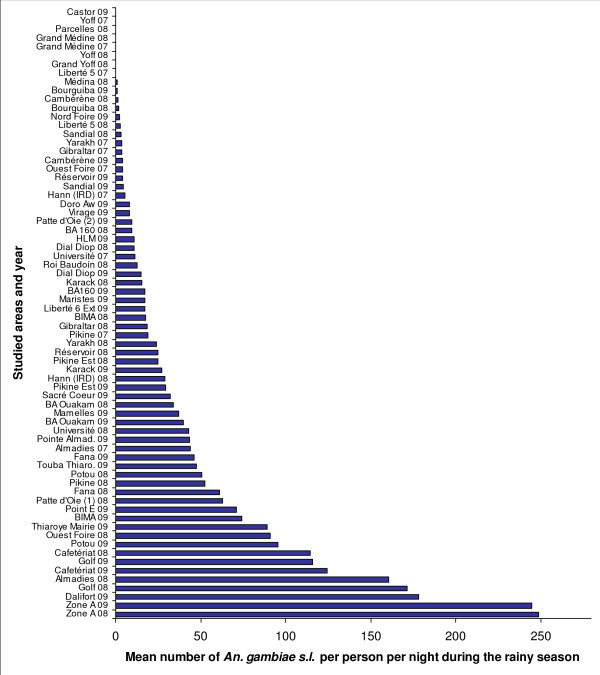
**Mean *An. gambiae s.l*. Rainy HBR for 45 studied areas in Dakar, for 2007, 2008 and 2009 rainy seasons (Sept-Oct)**.

**Figure 5:**
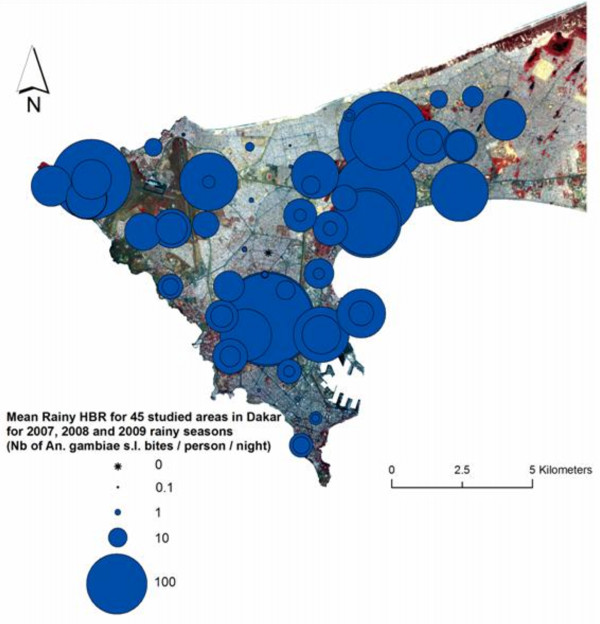
**Mean *An. gambiae s.l*. Rainy HBR for 45 studied areas in Dakar, for 2007, 2008 and 2009 rainy seasons (Sept-Oct), spatial distribution**.

**Figure 6:**
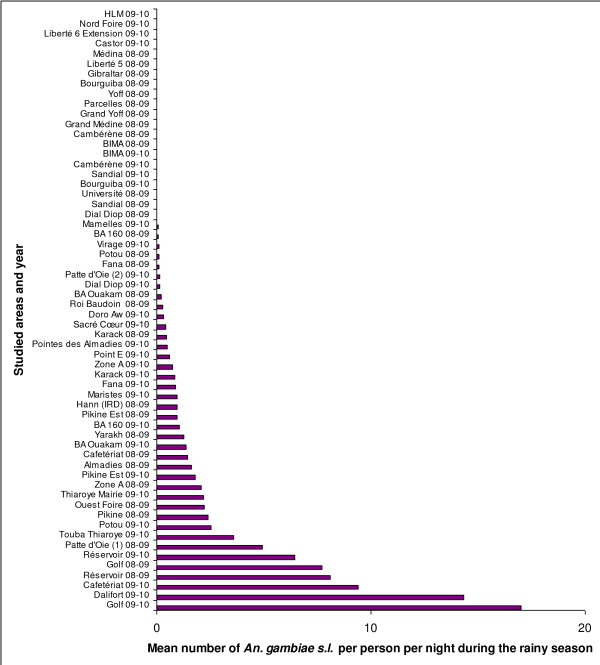
**Mean *An. gambiae s.l*. Dry HBR for the studied areas in Dakar, for 2008 and 2009 dry seasons (Nov-Jun)**.

Figure 3 shows that the peak of *An. gambiae s.l*. densities occurred between 15 to 20 days after the peak of precipitation. It also shows the high seasonality in the HBR during the year and a marked peak during the rainy season. In 2007, the mean *An. gambiae s.l*. Rainy HBR ranged from 0.1 bites per person per night in Yoff to 43.7 in Almadies. In 2008, the Rainy HBR value ranged from 0.3 bites per person per night in Grand Medine to 248.9 in Zone A, and in 2009, this value ranged from 0 bites per person per night in Castor to 244.8 in Zone A (Additional file 4). The highest recorded HBR was 713.5 bites by *An. gambiae s.l*. per person per night at one outdoors catching point on September 17, 2008 in Zone A. Figure 4 provides the mean Rainy HBR for every studied area, Figure 5 shows the spatial distribution of these Rainy HBR, and Figure 6 provides the mean Dry HBR for each zone. The highest *Anopheles *Dry HBR value reached more than 15 bites per person per night (Golf area).

A total of 44,628 *An. gambiae s.l*. were analysed by ELISA for the detection of the *P. falciparum *antigen, and 54 were positive (52 *An. arabiensis *and two *An. melas*). Table 4 presents the spatial and temporal distribution of the infected *Anopheles *that were found in 22 different studied areas. The average CSP index for all of the studied areas was 0.64% in the 2007 rainy season, 0.09% in 2008-2009 and 0.12% in 2009-2010. In the studied zones that were followed for more than one year, the CSP indexes were not significantly different between years. In 2008-2009 and 2009-2010, the annual CSP index was similar to the CSP indexes that were calculated for the mosquitoes that were caught during September-October. In consequence, the CSP index that was calculated in September-October of 2007 could be compared with the CSP indexes of 2008-2009 and 2009-2010 (Additional file 5). For 2007, 2008-2009 and 2009-2010, an average of 3.5, 2.3 and 3.4 infected bites, respectively, may have been received during the peaks of transmission (infected bites ranged from 0 to 17.6, depending on the studied areas and years).

**Table 4:** Spatial and temporal distribution of the *P. falciparum *infected *An. arabiensis *and *An. melas *caught in 45 studied areas in Dakar in Sept-Oct 2007, Jul 2008-Jun 2009 and Jul 2009-Jun 2010.

LOCALITE	Studied period* *Rainy/Dry*	Jul	Aug	Sept	Oct	Nov	Dec	Jan	Feb	Mar	Apr	May	Jun	Tot
Almadies	2007			3	1									4

Almadies	2008/09		1	1	1									3

Hann (IRD)	2008/09				1		1							2

Ouest Foire	2007				1									1

Ouest Foire	2008/09			3	1									4

Pikine	2007			1										1

Yarakh	2007			1										1

Patte d'Oie (1)	2008/09			2										2

BA Ouakam	2008/09		1											1

BA Ouakam	2009/10			1	1									2

Cafetériat	2009/10				5									5

Fana	2009/10				1									1

Golf	2008/09			1										1

Golf	2009/10							3			1		1	5

Karack	2008/09				1									1

Pikine Est	2008/09				1									1

Pikine Est	2009/10				1									1

Potou	2009/10				1									1

Réservoir	2008/09			1		1	1							3

Zone A	2008/09			1										1

Zone A	2009/10			1	3									4

Dalifort	2009/10			1				1						2

Liberté 6 Extension	2009/10				1									1

Mamelles	2009/10				1									1

Point E	2009/10				1									1

Sacré Cœur	2009/10			1		1								2

Thiaroye Mairie	2009/10				1									1

Touba Thiaroye	2009/10				1									1

Total			2	18	24	2	2	4			1		1	54

#### Correlation between larvae and adult densities

**Figure 7:**
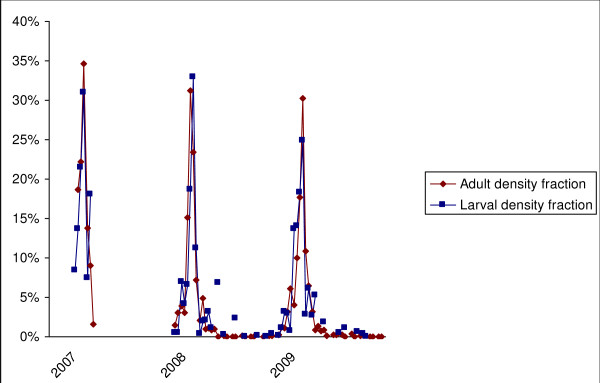
**Temporal variations of *Anopheles *adult density fraction and *Anopheles *larval density fraction**. Data collected in 45 studied areas in Dakar in Sept-Oct 2007 and Jul 2008-April 2010.

Figure 7 shows a very close parallel between larval and adult density fractions for the three studied years. In fact, the correlation coefficient between these fractions was 0.87.

## Discussion

### *Anopheles *larval habitats in Dakar

Several factors were found to be associated with the occurrence and abundance of anopheline larvae that were collected in Dakar from 2007 to 2010. Using multivariate analyses, the probability of the presence of anopheline larvae and the larval density were positively associated with numerous factors that could be classified into the following four categories: climatic factors (rainy season), environmental factors (lower level of urbanization), morphological factors (temporary collections) and biological factors (increased surface vegetation, higher water temperature, higher pH, and the absence of larvivorous fishes). These results were consistent with the known preference of *An. gambiae s.l*. for breeding in temporary pools [28,32]. Higher water temperature was not related to the presence of anopheline larvae but was linked to the density of larvae in a collection. The water temperature is perhaps not a key factor in the laying of eggs by parous females; however, it impacts the development of *An. gambiae *immature stages. Low-floating vegetation was previously found to be a determinant of the presence of anopheline larvae [12,33]; however, in the present study, the level of floating vegetation was positively associated with the presence of larvae. This difference could be explained by the difficulties of measuring the surface of vegetation that covers a water collection. Also, the surface vegetation can be a proxy for the presence of food, which can favour the presence of anopheline larvae. The pH was significantly associated with the occurrence or the abundance of larvae, and this factor could also be considered to be an indicator of the presence of food for larvae. The existence of a muddy bottom in and around the water collection was favourable for the presence of larvae, and this observation was consistent with the possibility for *An. gambiae *to lay their eggs on soil around puddle larval habitats [34,35]. However, a muddy bottom could also be a proxy for an increased persistence of water, which is different from asphalt or sand. As was previously described, the presence of predator fishes was associated with a lower probability of larvae or lower larval density. In 1998 in Dakar, Awono-Ambéné *et al *[36] confirmed that predation in the "céanes" was probably mainly due to fishes (Gambusia and Tilapia) that were introduced in Dakar in the 1930s for larval control. Many works have been conducted on the impact of urban agriculture on malaria transmission [11-15,37,38], and most of these studies have shown that this practice was associated with a higher level of malaria transmission in the surroundings areas. Specifically, urban agricultural activities can provide breeding and resting sites for malaria vectors. The results of the present study tend to show that market-gardens provided resting sites for adult *Anopheles*, rather than increased the number of larval habitats, as was previously demonstrated in Ghana [37]. Indeed, the water collections that were located in market-gardens less frequently harboured anopheline larvae, and when anopheline larvae were present, the densities were lower than in surroundings areas (as determined by univariate analysis). This occurrence was probably due to the presence of larvivorous fishes in traditional wells and to the frequent perturbations of water due to watering; however, one cannot exclude that this effect occurred because of the use of pesticides by urban farmers [39,40].

### *Anopheles *larvae in public and private areas

The present study has found that few larval habitats were located inside private sectors, in comparison to public areas (*i.e.*, freely accessible areas). Regardless of the type of housing or socio-economic level, the population generally did not allow *Anopheles *breeding sites to appear and persist in their neighbourhoods. As a consequence, the efficacy of any larval control measures would not suffer by being focused on freely accessible public zones, and excluding private areas from this treatment could simplify the control procedures.

### Spatial heterogeneity in malaria transmission in Dakar

The present results provide new evidence for malaria transmission in downtown Dakar and its nearby suburbs. The spatial heterogeneity of HBR was very marked, and it ranged from 0.1 to nearly 250 bites per person per night during the rainy season. According to the analysis undertaken in the studied areas, most of the adult *Anopheles *were caught between July and December, and a marked peak occurred in September-October. From 2007 to 2010, *An. gambiae s.l*. that had been infected with *P. falciparum *were caught in 22 of the 45 studied zones. Combined with the investigations that were conducted in 2005 and 2006 [6], it was determined that infected *An. gambiae s.l *have been caught in 24 of the 45 studied zones. Among the 14 areas where infected *An. gambiae s.l*. have been caught, no statistical difference has been highlighted between the CSP indexes for two consecutive years, indicating that no major change could have been detected in the infection levels in Dakar. Even if the CSP indexes had been calculated with the figures from the rainy season in 2007 and the annual figures in 2008-2009 and 2009-2010, it has been possible to compare data because the present study showed that the *An. gambiae s.l*. bites were concentrated in September and October, with a minimal HBR thereafter (constantly less than 5 bites per person per night from November to July). Consistency between the mosquito collections (and the HBR evaluation) was also ensured during the study period because the protocol for sampling remained unchanged. In addition, no major environmental changes have been recorded in the studied areas because of the low evolution rate in previously urbanised areas, which allows for the comparison of adult mosquito figures between years.

The ELISA method for the detection of CSP antigen in malaria vectors has been proven to detect more positive mosquitoes than dissection of salivary glands. However, the ELISA method is subject to less inter-operator variations and is less time-consuming than salivary gland dissection. For these reasons, ELISA is often considered to be the most reliable method for estimating the proportion of *Plasmodium*-infected malaria vectors and for analysing malaria transmission.

### *Anopheles *species involved in malaria transmission in Dakar

The following five anopheline species were collected from the 45 studied areas of Dakar: three species from the *An. gambiae s.l*. complex, *Anopheles pharoensis *and *Anopheles ziemanni*. *Anopheles ziemanni *had been previously caught on human bait at low levels in Senegal [41], as was the case in the present study, probably because of the low affinity of this species to human blood. *Anopheles pharoensis *is considered to be a secondary vector for malaria and has been found in the present study at low density. The presence of *An. gambiae s.s. M form *was reported in 2008-2009 (and in 2005-2006 in [6]) at extremely low levels. Therefore, *An. gambiae s.s., An. ziemanni *and *An. pharoensis *could not be significantly involved in malaria transmission in Dakar.

From 2007 to 2010 in the 45 studied zones, 54 *An. gambiae s.l*. were infected by *P. falciparum*: 52 *An. arabiensis *and two *An. melas*. Both infected *An. melas *were caught in Golf, near the large marshy area, in 2010, and this was the first time that this species was found to be infected with *P. falciparum *in Dakar. From 2007 to 2010, *An. melas *has been reported in 18 zones, where it accounted for 1% to 40.9% of the *An. gambiae *complex, according to the area and the year. Because *An. melas *is not as good a vector as *An. arabiensis *[42], it could be involved in malaria transmission only in areas in Dakar where it is very abundant. None of the breeding sites were exclusive for *An. melas *during the entire duration of the survey, and *An. arabiensis *was always found at these sites on at least one date, probably because of variations in the salinity levels of the breeding habitats. The co-occurrence of both of these species indicates that *An. arabiensis *larvae may also have a tolerance for low salinity.

### Biting behaviours of adult *Anopheles*

In 2007, 2008-2009 and 2009-2010, 21%, 30% and 26%, respectively, of *An. gambiae s.l*. were caught indoors (at one indoor versus two outdoor catching points), but it was not possible to define a specific behaviour according to the season or to the studied area. Because logistical constraints limited the number of indoor catching points to one per site and the characteristics of the rooms could be different between areas (*e.g.*, public offices or private properties of different sizes and types), the experimental design may have not allowed for the results to be representative of the adult *An. gambiae s.l*. behaviour in each of the studied areas. It can only be concluded that malaria transmission can occur in Dakar both outdoors and indoors. No difference was recorded in the hours of activity of *An. gambiae s.l*. between the different studied sites, and most of *An. gambiae s.l*. bites (> 75%) were recorded to take place after midnight. This finding highlighted the interest of using insecticide-impregnated bed nets to control malaria transmission in Dakar. Nevertheless, some bites were received during the first hours of the night when people were generally not asleep and not protected by mosquito nets. The use of other mosquito-control devices, like repellents or mosquito coils, could be proposed to increase protection for people from mosquitoes during the first hours of the night in Dakar.

### Temporal heterogeneity of malaria transmission in Dakar

The abundance of *An. gambiae s.l*. during the year was clearly dependent on rainfall because the peak of the *An. gambiae s.l*. biting rate followed the peak of rainfall, with a two-week lag, regardless of the year. Afterward, the densities quickly decreased after the rains ended, and only isolated specimens were caught until the beginning of the new rainy season in July. Nevertheless, in one area (Golf), some infected mosquitoes were caught during the dry season, indicating that a permanent malaria transmission is possible in this area of Dakar, which is located near the large marshy area. When comparing the human biting rates in the common areas between 2007, a year with low rainfall, and 2008, a year with heavy rainfall, the peaks of *Anopheles *bites were, on average, six-fold higher in 2008 than in 2007. This was consistent with the fact that rainfall is closely related to the presence of surface water, larval productivity and adult densities, the latter two being highly correlated. Indeed, several studies have highlighted the low dispersion of *Anopheles *in urban settings due to the high density of houses and the proximity of readily available hosts for blood meals; therefore, most *An. gambiae s.l*. that are caught on human bait probably hatched from eggs that have been laid in a breeding site in the same or neighbouring area [15-19].

### Toward malaria risk mapping

Most of the factors that are associated with the presence and density of larvae in water collections are measurable or detectable, either directly or indirectly, by remote sensing (RS) and include the following: the soil temperature, vegetation and trees that are able to provide shade, the surface vegetation, the moisture level of the ground, the density of urbanization, and the persistence of water collections during the dry season. Consequently, RS and geographic information systems (GIS) can provide useful information for mapping *Anopheles *breeding sites, as has been shown in numerous studies [43]. Furthermore, the close relationship between larval density and adult HBR suggests that maps of *Anopheles *breeding sites could provide baseline data for mapping *Anopheles *adult densities.

## Conclusion

Suitable conditions for malaria transmission in Dakar were present during the extended rainy season from July to December, and the *An. gambiae s.l*. HBR increased with the intensity of rainfall in most of the studied areas. Nevertheless, the probability of encountering malaria vectors was not the same between the different areas. Taking into account this highly focused risk of malaria transmission, a targeted approach of vector control must be chosen. Because they are limited, the financial, material and human resources that are available must be concentrated on areas with a higher risk of transmission. Because it would not be reasonable to conduct routine, entomological studies in the entire city of Dakar, efforts should be concentrated on drawing risk maps to help targeting those anti-vectorial activities. In conclusion, strong, spatial and temporal heterogeneity of *An. gambiae s.l*. larval density, HBR and malaria transmission in Dakar has been confirmed, and the environmental factors that are associated with this heterogeneity have been identified. Moreover, the results of the present study will pave the way to set up malaria risk maps using RS and GIS technologies.

## Competing interests

The authors declare that they have no competing interests.

## Authors' contributions

LG was responsible for the supervision of data collection, analysis, interpretation, production of the final manuscript and revisions. VM was responsible for the supervision of data collection, analysis, interpretation, production of the final manuscript and revisions. FP was responsible for overall scientific management, analysis, interpretation, production of the final manuscript and revisions. AG contributed to the supervision of data collection and data analysis. FJ contributed to the data analysis. LGo contributed to the data analysis. BC contributed to the data collection. JPL contributed to overall scientific management. CS contributed to overall scientific management. JFT contributed to overall scientific management. CR conceived and designed the study and was responsible for overall scientific management, analysis, interpretation, preparation of the final manuscript and revisions. All authors read and approved the final manuscript.

## Supplementary Material

Additional file 1 **Description of the quantitative physical, biological and chemical parameters recorded for the open water collections in 45 studied areas in Dakar in October-September 2007 and between July 2008 and April 2010, depending on breeding status**. Click here for file

Additional file 2 **Factors associated with the presence/absence of *Anopheles *larvae and *Anopheles *larval densities recorded in the open water collections among 45 studied areas in Dakar in October-September 2007 and between July 2008 and April 2010**. Logistic regression and binomial negative regression with water collection random effect. Univariate analysis. Click here for file

Additional file 3 **Numbers and proportions of species among the *An. gambiae s.l*. collected on humans in 45 studied areas in Dakar in Sept-Oct 2007 and Jul 2008-Jun 2010**. Click here for file

Additional file 4 ***Anopheles gambiae s.l*. HBR and rainfall events in 10 studied areas in September-October 2007, 30 studied areas in July 2008 - June 2009 and 30 studied areas in July 2009 - June 2010**. Details for each studied area. Click here for file

Additional file 5 **Annual CSP index of each studied area, annual CSP index averaged for all studied areas and *An. gambiae s.l*. Rainy EIR (Sept-Oct) for each of the 45 studied areas in Dakar in Sept-Oct 2007 and Jul 2008-Jun 2010**. Click here for file
