# Supplementary material for: Conditions of malaria transmission in Dakar from 2007 to 2010
Source: Malar J. 2011 Oct 21;10:312. doi: 10.1186/1475-2875-10-312 (PMC3216462; doi:10.1186/1475-2875-10-312)
Supplement: Additional file 1 — Description of the quantitative physical, biological and chemical parameters recorded for the open water collections in 45 studied areas in Dakar in October-September 2007 and between July 2008 and April 2010, depending on breeding status. [file 1475-2875-10-312-S1.PDF]

Additional file 1. Description of the quantitative physical, biological and chemical parameters recorded for the open water collections in 45 studied areas in Dakar in October-September 2007 and between July 2008 and April 2010, depending on breeding status.

**Parameters**	**Anopheline larvae and pupae absent**	**Anopheline larvae and pupae present**
**Continuous variables**	**Number of observations**	**Number of observations**
	**Range**	**Range**
	**Mean and 95% Confidence Interval**	**Mean and 95% Confidence Interval**
Surface (m²)	1014	1657
	0.01 - 30550	0.01 - 50662
	1861 [1613 - 2109]	2190 [1797 - 2583]
Water temperature (°C)	1 430	857
	18.7 - 42.3	19.1 - 41.2
	29.0 [28.8 - 29.2]	30.7 [30.5 – 31.0]
Turbidity (from 0: clear, to 26: turbid)	1597	979
	0 - 26	0 - 26
	9.8 [9.3 – 10.3]	11.9 [11.2 – 12.5]
pH	1609	985
	4.2 - 10.1	4.4 - 11.3
	7.6 [7.6 - 7.7]	8.1 [8.1 - 8.2]
Conductivity (not recorded in 2007)	935	559
	0.02 – 20.00	0.04 – 20.00
	3.00 [2.78 – 3.21]	3.03 [2.74 – 3.32]
Shade (%)	1664	1012
	0 - 100	0 - 100
	30.4 [28.8 – 32.0]	19.4 [17.6 – 21.2]
Surface vegetation (%)	1638	1013
	0 - 100	0 - 100
	34.0 [32.1 – 35.9]	27.7 [25.7 – 29.6]
Number of continuous decades of water persistence	502	577
	1 - 10	1 - 9
	2.7 [2.5 – 2.9]	3.4 [3.3 – 3.6]

**Parameters**		**Anopheline larvae and pupae absent**	**Anopheline larvae and pupae present**
**Categorical variables**		**Number of observations (row %)**	**Number of observations (row %)**
Presence of *Culicinae* larvae			
	No	1376 (76.2%)	430 (23.8%)
	Yes	284 (33.5%)	563 (66.5%)
Presence of any predator, including larvivorous fishes			
	No	665 (66.2%)	340 (33.8%)
	Yes	819 (60.0%)	546 (40.0%)
Presence of larvivorous fishes			
	No	1037 (56.2%)	808 (43.8%)
	Yes	445 (85.4%)	76 (14.6%)
Season			
	Dry (Nov to Jun)	575 (78.4%)	185 (21.6%)
	Wet (Jul to Oct)	1093 (56.1%)	857 (43.9%)
Studied year			
	Jul 2008 - Jun 2009	658 (59.2%)	453 (40.8%)
	Jul 2009 - Jun 2010	826 (65.6%)	433 (34.4%)
Persistence of water collection			
	Temporary	772 (49.9%)	776 (50.1%)
	Permanent	896 (78.9%)	239 (21.1%)
Type of water collection			
	Canal	58 (63.7%)	33 (36.3%)
	Ditch or puddle	496 (48.1%)	535 (51.9%)
	Lake, pond, marshland	255 (61.7%)	158 (38.3%)
	Well or basin	793 (80.4%)	193 (19.6%)
	Small containers	11 (44.0%)	14 (56%)
	Hole	55 (40.1%)	82 (59.9%)
Type of water collection (puddle vs others)			
	Ditch or puddle	496 (48.1%)	535 (51.9%)
	Other	1172 (70.9%)	480 (29.1%)

**Parameters**		**Anopheline larvae and pupae absent**	**Anopheline larvae and pupae present**
**Categorical variables**		**Number of observations (row %)**	**Number of observations (row %)**
Muddy bottom			
	No	1423 (67.9%)	674 (32.1%)
	Yes	238 (41.2%)	340 (58.8%)
Water collection located in market-garden			
	No	944 (51.3%)	896 (48.7%)
	Yes	724 (85.9%)	119 (14.1%)
Water collection located in highly urbanized area			
	No	1601 (61.4%)	1008 (38.6%)
	Yes	67 (90.5%)	7 (9.5%)

Parameters	Anopheline larvae and pupae absent	Anopheline larvae and pupae present
**Categorical variables**	**Number of observations (row %)**	**Number of observations (row %)**
Studied area		
Almadies	26 (25.7%)	75 (74.3%)
BA Ouakam	19 (29.2%)	46 (70.8%)
BA160	40 (71.4%)	16 (28.6%)
BIMA	13 (48.1%)	14 (51.9%)
Bourguiba	26 (92.9%)	2 (7.1%)
Cafetériat	17 (38.6%)	27 (61.4%)
Cambérène	11 (78.6%)	3 (21.4%)
Dalifort	119 (79.9%)	30 (20.1%)
Dial Diop	49 (41.2%)	70 (58.8%)
Fana	24 (44.4%)	30 (55.6%)
Gibraltar	6 (33.3%)	12 (66.7%)
Golf	155 (66.8%)	77 (33.2%)
Grand Médine	10 (66.7%)	5 (33.3%)
Grand Yoff	3 (100.0%)	0 (0.0%)
Hann (IRD)	37 (46.2%)	43 (53.8%)
HLM	1 (100.0%)	0 (0.0%)
Karack	35 (46.1%)	41 (53.9%)
Liberté 5	9 (69.2%)	4 (30.8%)
Liberté 6 Extension	25 (78.1%)	7 (21.9%)
Mamelles	8 (34.8%)	15 (62.2%)
Maristes	62 (87.3%)	9 (12.7%)
Nord Foire	2 (100.0%)	0 (0.0%)
Ouest Foire	37 (29.8%)	87 (70.2%)
Patte d'Oie (1)	72 (83.7%)	14 (16.3%)
Patte d'Oie (2)	95 (83.3%)	19 (16.7%)
Pikine	174 (72.5%)	66 (27.5%)
Pikine Est	5 (83.3%)	1 (16.7%)
Point E	9 (100.0%)	0 (0.0%)
Pointe des Almadies	33 (64.7%)	18 (35.3%)
Potou	36 (59.0%)	25 (41.0%)
Réservoir	80 (70.2%)	34 (29.8%)
Roi Baudoin	37 (88.1%)	5 (11.9%)
Sacré Cœur	16 (41.0%)	23 (59.0%)
Sandial	0	0
Thiaroye Mairie	90 (75.6%)	29 (24.4%)
Touba Thiaroye	17 (73.9%)	6 (26.1%)
Université	16 (29.1%)	39 (70.9%)
Virage	25 (65.8%)	13 (34.2%)
Yarakh	191 (87.6%)	27 (12.4%)
Zone A	38 (31.4%)	83 (68.6%)
