# Supplementary material for: Conditions of malaria transmission in Dakar from 2007 to 2010
Source: Malar J. 2011 Oct 21;10:312. doi: 10.1186/1475-2875-10-312 (PMC3216462; doi:10.1186/1475-2875-10-312)
Supplement: Additional file 2 — Factors associated with the presence/absence of Anopheles larvae and Anopheles larval densities recorded in the open water collections among 45 studied areas in Dakar in October-September 2007 and between July 2008 and April 2010. Logistic regression and binomial negative regression with water collection random effect. Univariate analysis. [file 1475-2875-10-312-S2.PDF]

Additional file 2. Factors associated with the presence/absence of *Anopheles* larvae and *Anopheles* larval densities recorded in the open water collections among 45 studied areas in Dakar in October-September 2007 and between July 2008 and April 2010. Logistic regression and binomial negative regression with water collection random effect. Univariate analysis.

	Presence/absence of *Anopheles* larvae				*Anopheles* larval density			
	Nb of observations	OR	95% CI	p-value	Nb of observations	IRR	95% CI	p-value
Surface	2 671				1 008			
<2 000 m²		1				1		
>=2 000 m²		1.09	0.58 - 2.02	0.795		0.73	0.62 - 0.87	0.0005
Water temperature (°C)	2 287				857			
<30°C		1				1		
>=30°C		1.69	1.31 - 2.16	<0.0001		1.31	1.16 - 1.47	<0.0001
Turbidity (from 0: clear, to 26: turbid)	2 576				975			
Clear (<10)		1				1		
Turbid (>=10)		1.30	1.01 - 1.67	0.042		1.03	0.92 - 1.16	0.574
pH	2 594				980			
<8		1				1		
>=8		1.89	1.46 - 2.45	<0.0001		1.27	1.13 - 1.43	0.0001
Conductivity (not recorded in 2007)	1 494				557			
<3		1				1		
>=3		0.80	0.59 - 1.10	0.167		0.91	0.78 - 1.06	0.228
Shade (%)	2 676				1 005			
<20%		1				1		
>=20%		0.62	0.41 - 0.94	0.023		0.82	0.71 - 0.94	0.004
Surface vegetation (%)	2 651				1 008			
<20%		1				1		
>=20%		1.68	1.22 - 2.32	0.001		0.92	0.82 - 1.04	0.196
Presence of *Culicinae* larvae	2 653				993			
No		1				1		
Yes		13.57	9.94 - 18.53	<0.0001		0.94	0.84 - 1.06	0.342
Presence of larvivorous fishes	2 366				882			
No		1				1		
Yes		0.35	0.22 - 0.54	<0.0001		0.63	0.50 - 0.79	0.0001
Season	2 683				1 008			
Dry (Nov to Jun)		1				1		
Wet (Jul to Oct)		3.20	2.43 - 4.21	<0.0001		1.48	1.27 - 1.73	<0.0001
Persistence of water collection	2 683				1 008			
Permanent		1				1		
Temporary		10.25	5.63 - 18.63	<0.0001		1.52	1.29 - 1.79	<0.0001
Number of continuous decades of water persistence	1 079				575			
For every decade		1.21	1.12 - 1.31	<0.0001		1.00	0.96 - 1.03	0.734
Type of water collection (puddle vs others)	2 683							
Other		1				1		
Ditch or puddle		3.42	2.01 - 5.83	<0.0001		1.40	1.22 - 1.60	<0.0001

Muddy bottom		2 675				1 007			
	No		1				1		
	Yes		2.57	1.50 - 4.40	0.0006		1.24	1.07 - 1.44	0.003
Water collection located in market-garden		2 683				1 008			
	No		1				1		
	Yes		0.09	0.05 - 0.17	<0.0001		0.73	0.59 - 0.89	0.003
Water collection located in highly urbanized area		2 683				1 008			
	No		1				1		
	Yes		0.08	0.02 - 0.33	0.001		0.83	0.40 - 1.71	0.613

Additional file 2. Factors associated with the presence/absence of *Anopheles* larvae and *Anopheles* larval densities recorded in the open water collections among 45 studied areas in Dakar in October-September 2007 and between July 2008 and April 2010. Logistic regression and binomial negative regression with water collection random effect. Univariate analysis.
