# Supplementary material for: Conditions of malaria transmission in Dakar from 2007 to 2010
Source: Malar J. 2011 Oct 21;10:312. doi: 10.1186/1475-2875-10-312 (PMC3216462; doi:10.1186/1475-2875-10-312)
Supplement: Additional file 3 — Numbers and proportions of species among the An. gambiae s.l. collected on humans in 45 studied areas in Dakar in Sept-Oct 2007 and Jul 2008-Jun 2010. [file 1475-2875-10-312-S3.PDF]

	Sept-Oct 2007 (10 areas)			Jul 2008-Jun 2009 (30 areas)				Jul 2009-Jun 2010 (30 ares)		
Studied areas	*An. arabiensis* (% of *An. arabiensis*)	*An. melas* (% of *An. melas*)	Total number of specimens processed	*An. arabiensis* (% of *An. arabiensis*)	*An. melas* (% of *An. melas*)	*An. gambiae s.s M form* (% of *An. gambiae s.s M form*)	Total number of specimens processed	*An. arabiensis* (% of *An. arabiensis*)	*An. melas* (% of *An. melas*)	Total number of specimens processed
Almadies	102 (100%)		102	99 (100%)			99			
Pikine	78 (78.8%)	21 (21.2%)	99	71 (72.4%)	27 (27.6%)		98			
Université	93 (100%)		93	110 (100%)			110			
Hann (IRD)	58 (92.1%)	4 (6.4%)	62	68 (59.1%)	47 (40.9%)		115			
Ouest Foire	44 (100%)		44	119 (96%)	5 (4.0%)		124			
Yarakh	41 (100%)		41	105 (92.1%)	9 (7.9%)		114			
Gibraltar	43 (100%)		43	104 (100%)			104			
Liberté 5	7 (100%)		7	31 (100%)			31			
Grand Médine	3 (75.0%)	1 (25.0%)	4	6 (100%)			6			
Yoff	1 (100%)		1	7 (100%)			7			
Parcelles				2 (100%)			2			
Grand Yoff				8 (100%)			8			
Médina				9 (100%)			9			
Cambérène				11 (68.8%)	5 (31.2%)		16			
Bourguiba				21 (100%)			21			
Sandial				62 (100%)			62			
BA 160				93 (100%)			93			
Dial Diop				98 (99.0%)	1 (1.0%)		99			
Roi Baudoin				101 (100%)			101			
Karack				96 (99.0%)	1 (1.0%)		97			
BIMA				91 (97.8%)	2 (2.2%)		93			
Réservoir				67 (100%)			67			
Pikine Est				81 (100%)			81	1 (100%)		1
BA Ouakam				99 (100%)			99	1 (100%)		1
Potou				94 (95.9%)	4 (4.1%)		98	1 (100%)		1
Fana				99 (100%)			99	25 (100%)		25
Patte d'Oie (1)				110 (80.9%)	25 (18.4%)	1 (0.7%)	136			
Cafétéria				102 (91.9%)	9 (8.1%)		111	4 (100%)		4
Golf				108 (69.7%)	47 (30.3%)		155	3 (60.0%)	2 (40.0%)	5
Zone A				103 (73.6%)	37 (26.4%)		140	3 (100%)		3
Castor								0	0	0
Nord Foire								6 (100%)		6

	Sept-Oct 2007 (10 areas)			Jul 2008-Jun 2009 (30 areas)				Jul 2009-Jun 2010 (30 ares)		
Studied areas	*An. arabiensis* (% of *An. arabiensis*)	*An. melas* (% of *An. melas*)	Total number of specimens processed	*An. arabiensis* (% of *An. arabiensis*)	*An. melas* (% of *An. melas*)	*An. gambiae s.s M form* (% of *An. gambiae s.s M form*)	Total number of specimens processed	*An. arabiensis* (% of *An. arabiensis*)	*An. melas* (% of *An. melas*)	Total number of specimens processed
Doro Aw								4 (100%)		4
Virage								28 (100%)		28
Patte d'Oie (2)								33 (100%)		33
HLM								13 (76.5%)	4 (23.5%)	17
Liberté 6 Extension								55 (100%)		55
Marsites								33 (94.3%)	2 (5.7%)	35
Sacré Coeur								84 (97.7%)	2 (2.3%)	86
Mamelles								128 (100%)		128
Pointe de Almadies								92 (100%)		92
Touba Thiaroye								33 (100%)		33
Point E								103 (97.2%)	3 (2.8%)	106
Thiaroye Mairie								103 (100%)		103
Dalifort								118 (100%)		118

Additional 3. Numbers and proportions of species among the *An. gambiae s.l.* collected on humans in 45 studied areas in Dakar in Sept-Oct 2007 and Jul 2008-Jun 2010.
