# Supplementary material for: Conditions of malaria transmission in Dakar from 2007 to 2010
Source: Malar J. 2011 Oct 21;10:312. doi: 10.1186/1475-2875-10-312 (PMC3216462; doi:10.1186/1475-2875-10-312)
Supplement: Additional file 4 — Anopheles gambiae s.l. HBR and rainfall events in 10 studied areas in September-October 2007, 30 studied areas in July 2008 - June 2009 and 30 studied areas in July 2009 - June 2010. Details for each studied area. [file 1475-2875-10-312-S4.PDF]

Additional file 4: The high seasonality and the great heterogeneity of HBR during the year, with a marked peak during the rainy season in 45 zones in Dakar.

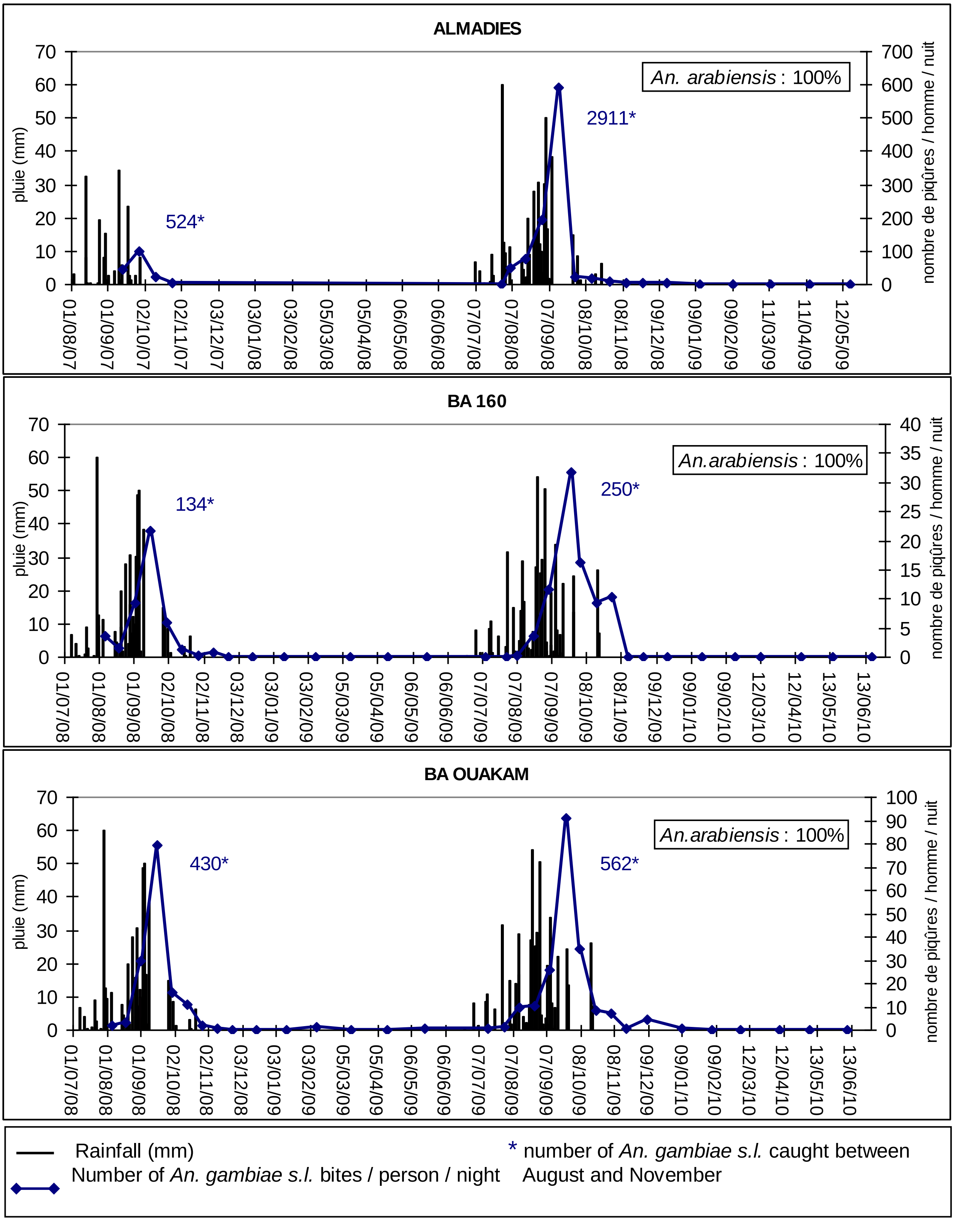

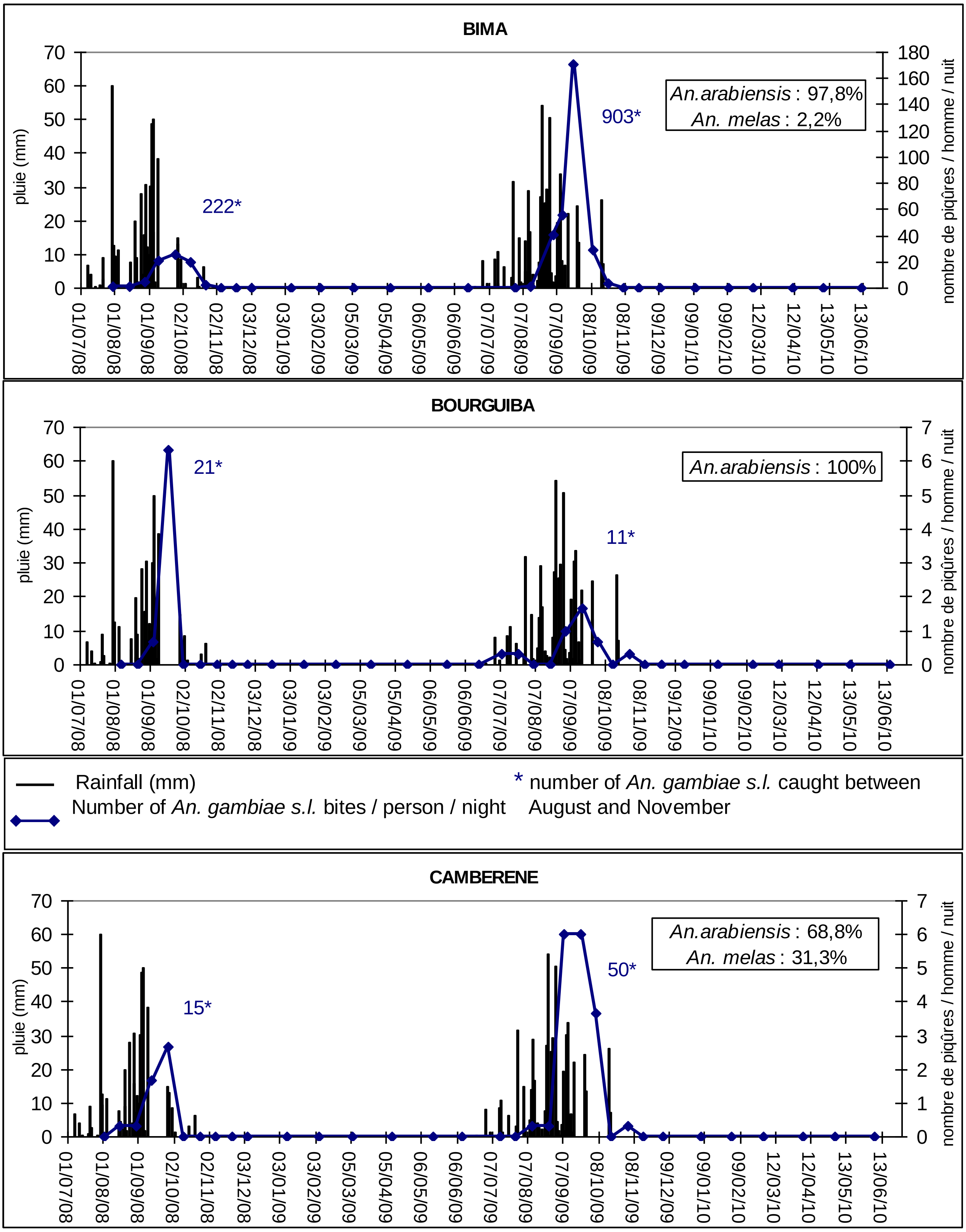
BIMA
pluie (mm)
nombre de piqûres / homme / nuit
An.arabiensis : 97,8%
An. melas : 2,2%
222*
903*
01/07/08
01/08/08
01/09/08
02/10/08
02/11/08
03/12/08
03/01/09
03/02/09
05/03/09
05/04/09
06/05/09
06/06/09
07/07/09
07/08/09
07/09/09
08/10/09
08/11/09
09/12/09
09/01/10
09/02/10
12/03/10
12/04/10
13/05/10
13/06/10
BOURGUIBA
An.arabiensis : 100%
21*
11*
Rainfall (mm)
Number of An. gambiae s.l. bites / person / night
* number of An. gambiae s.l. caught between August and November
CAMBERENE
An.arabiensis : 68,8%
An. melas : 31,3%
15*
50*

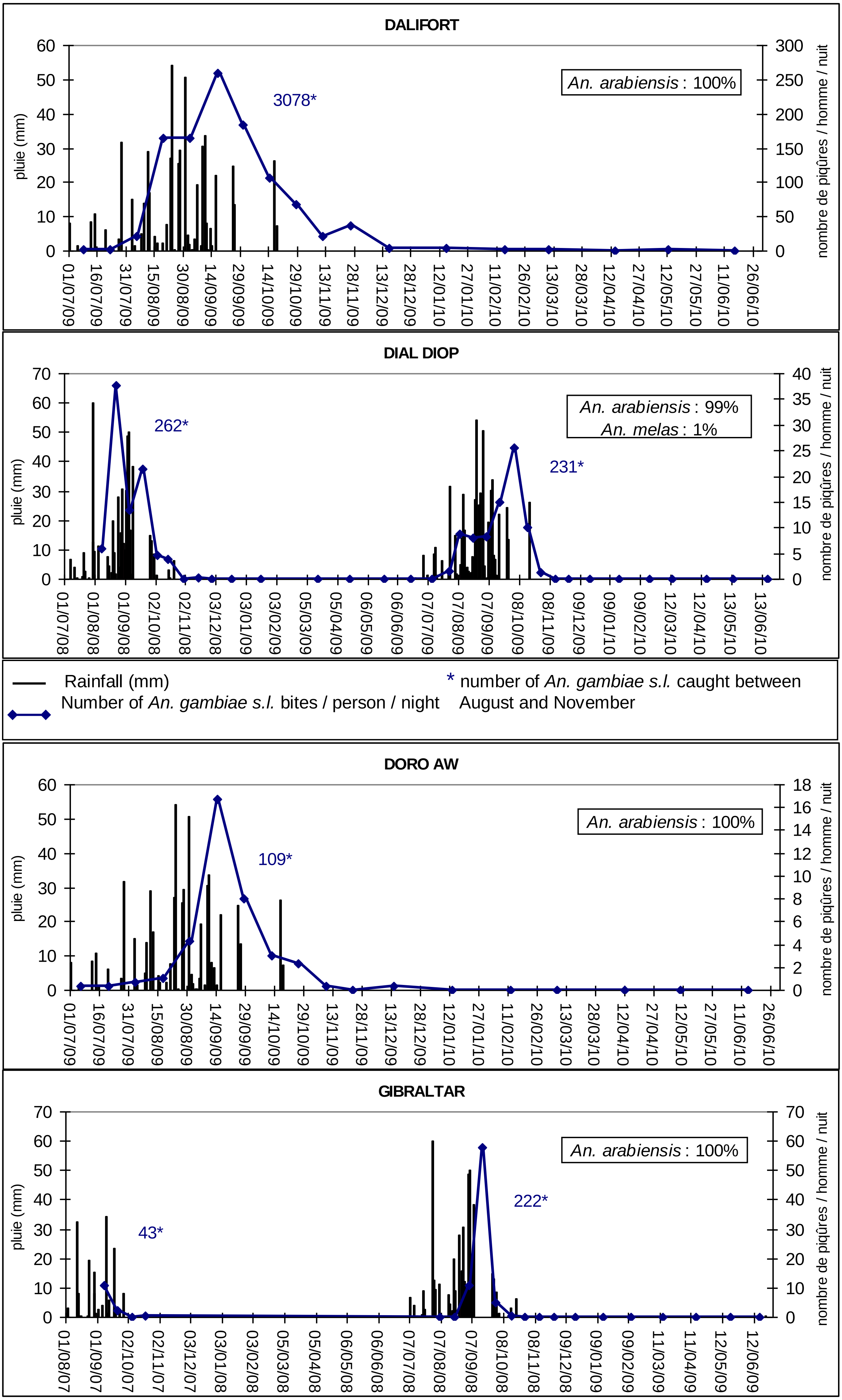

DALIFORT
pluie (mm)
nombre de piqûres / homme / nuit
An. arabiensis : 100%
3078*
DIAL DIOP
An. arabiensis : 99%
An. melas : 1%
262*
231*
Rainfall (mm)
Number of An. gambiae s.l. bites / person / night
* number of An. gambiae s.l. caught between August and November
DORO AW
An. arabiensis : 100%
109*
GIBRALTAR
An. arabiensis : 100%
43*
222*

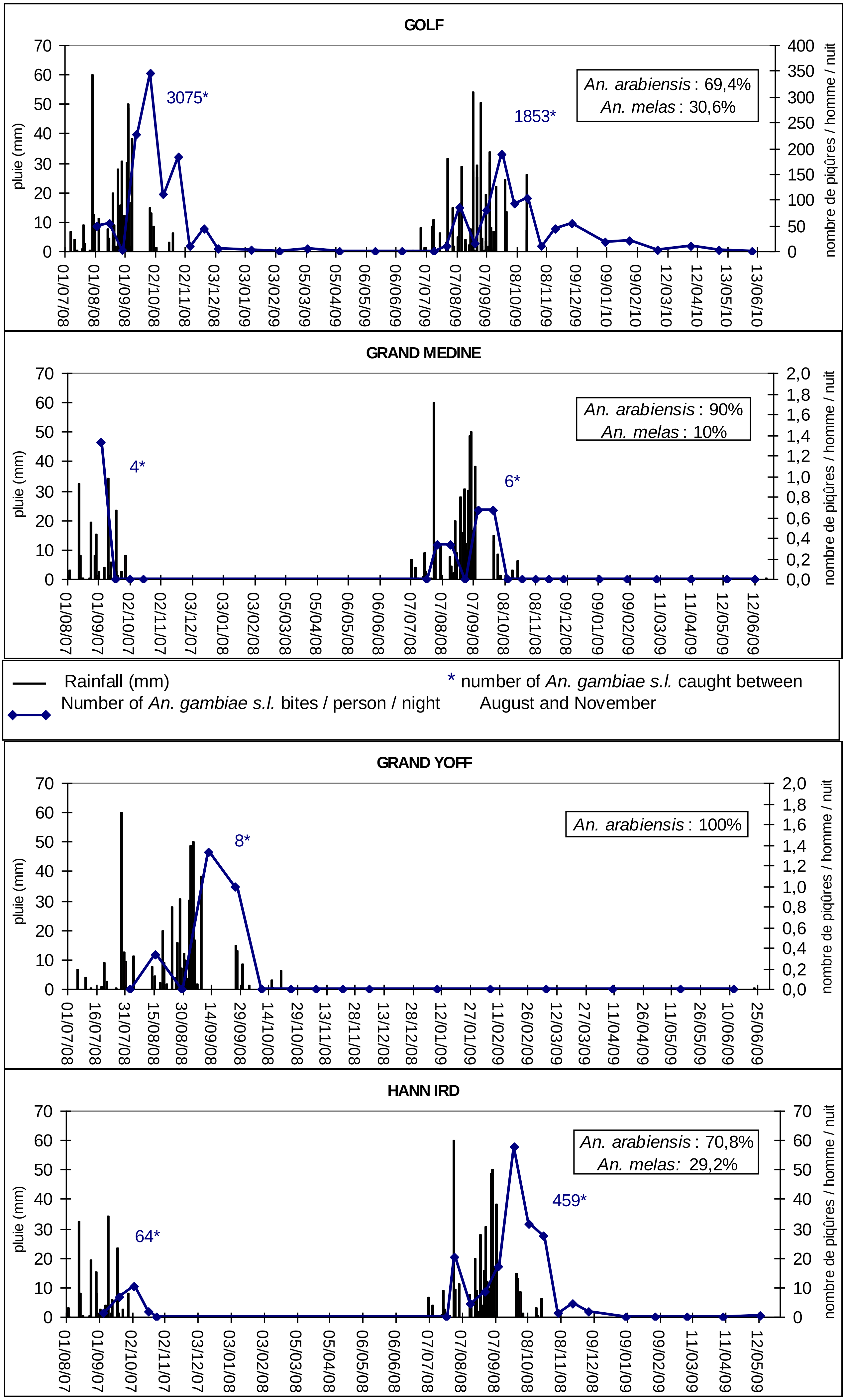
GOLF
An. arabiensis : 69,4%
An. melas : 30,6%
3075*
1853*
pluie (mm)
nombre de piqûres / homme / nuit
GRAND MEDINE
An. arabiensis : 90%
An. melas : 10%
4*
6*
pluie (mm)
nombre de piqûres / homme / nuit
Rainfall (mm)
Number of An. gambiae s.l. bites / person / night
* number of An. gambiae s.l. caught between August and November
GRAND YOFF
An. arabiensis : 100%
8*
pluie (mm)
nombre de piqûres / homme / nuit
HANN IRD
An. arabiensis : 70,8%
An. melas: 29,2%
64*
459*
pluie (mm)
nombre de piqûres / homme / nuit

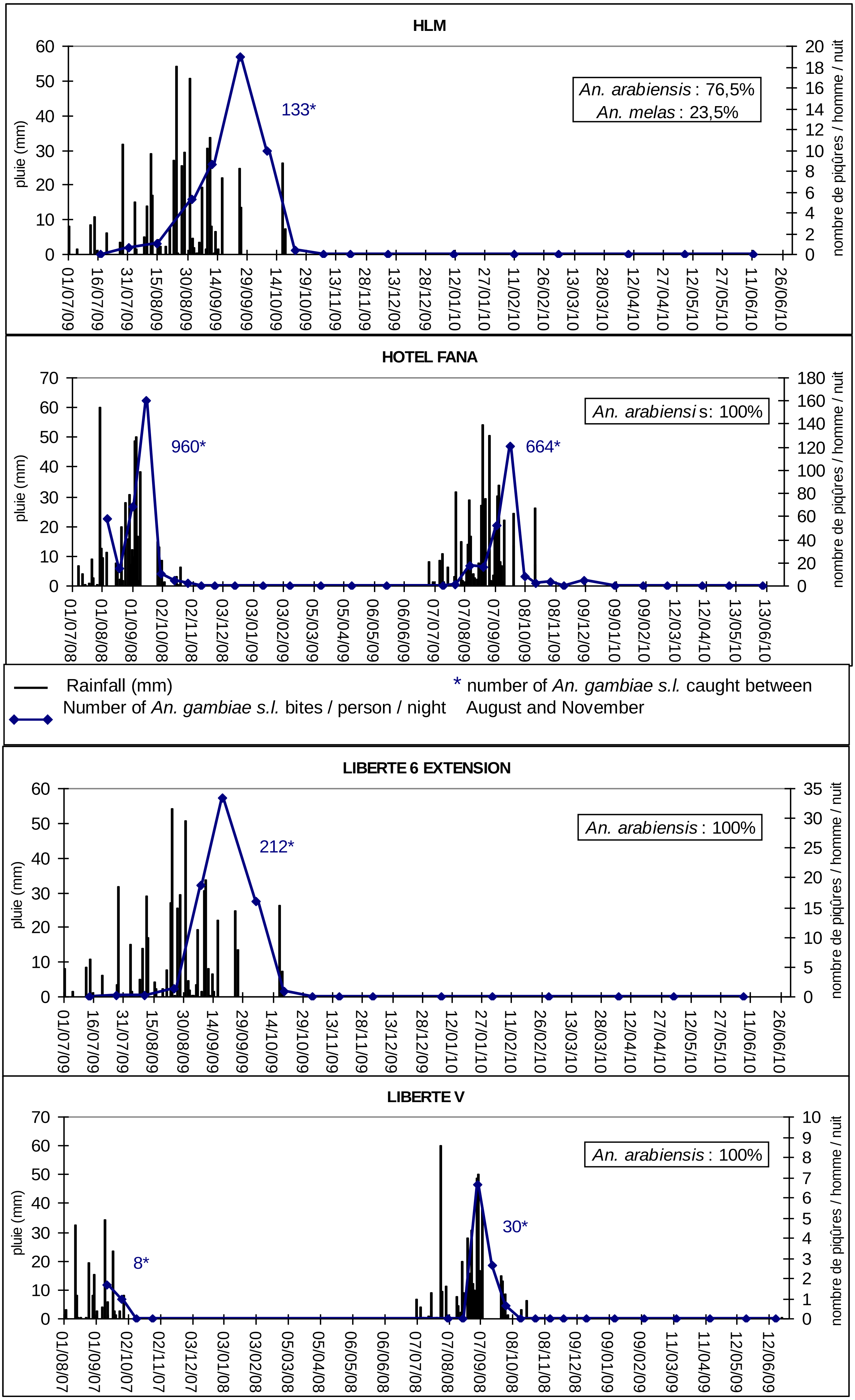
HLM
pluie (mm)
nombre de piqûres / homme / nuit
An. arabiensis : 76,5%
An. melas : 23,5%
133*
HOTEL FANA
An. arabiensi s: 100%
960*
664*
Rainfall (mm)
Number of *An. gambiae s.l.* bites / person / night
* number of *An. gambiae s.l.* caught between August and November
LIBERTE 6 EXTENSION
An. arabiensis : 100%
212*
LIBERTE V
An. arabiensis : 100%
8*
30*

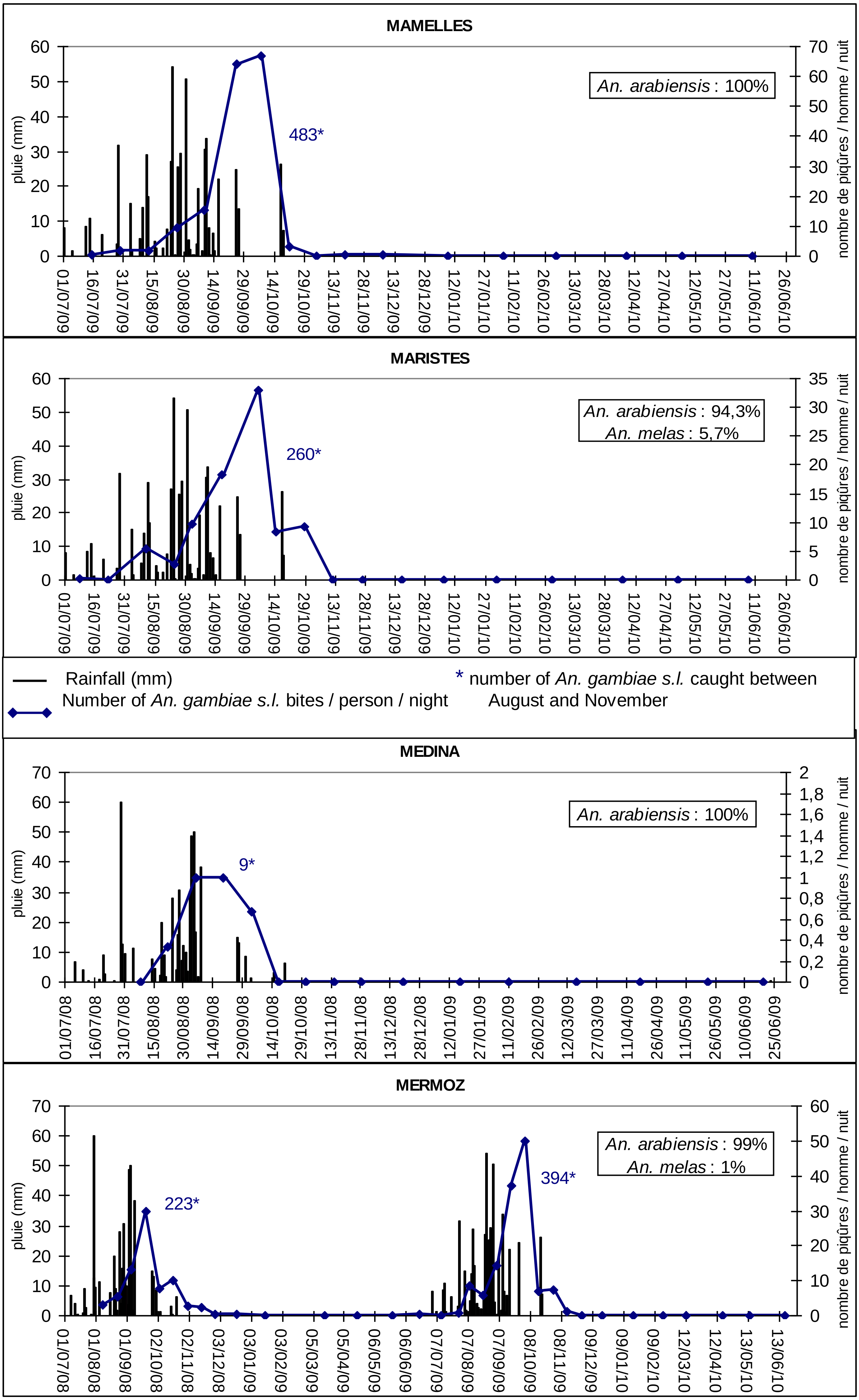

MAMELLES
pluie (mm)
nombre de piqûres / homme / nuit
An. arabiensis : 100%
483*
MARISTES
An. arabiensis : 94,3%
An. melas : 5,7%
260*
Rainfall (mm)
Number of An. gambiae s.l. bites / person / night
* number of An. gambiae s.l. caught between August and November
MEDINA
An. arabiensis : 100%
9*
MERMOZ
An. arabiensis : 99%
An. melas : 1%
223*
394*

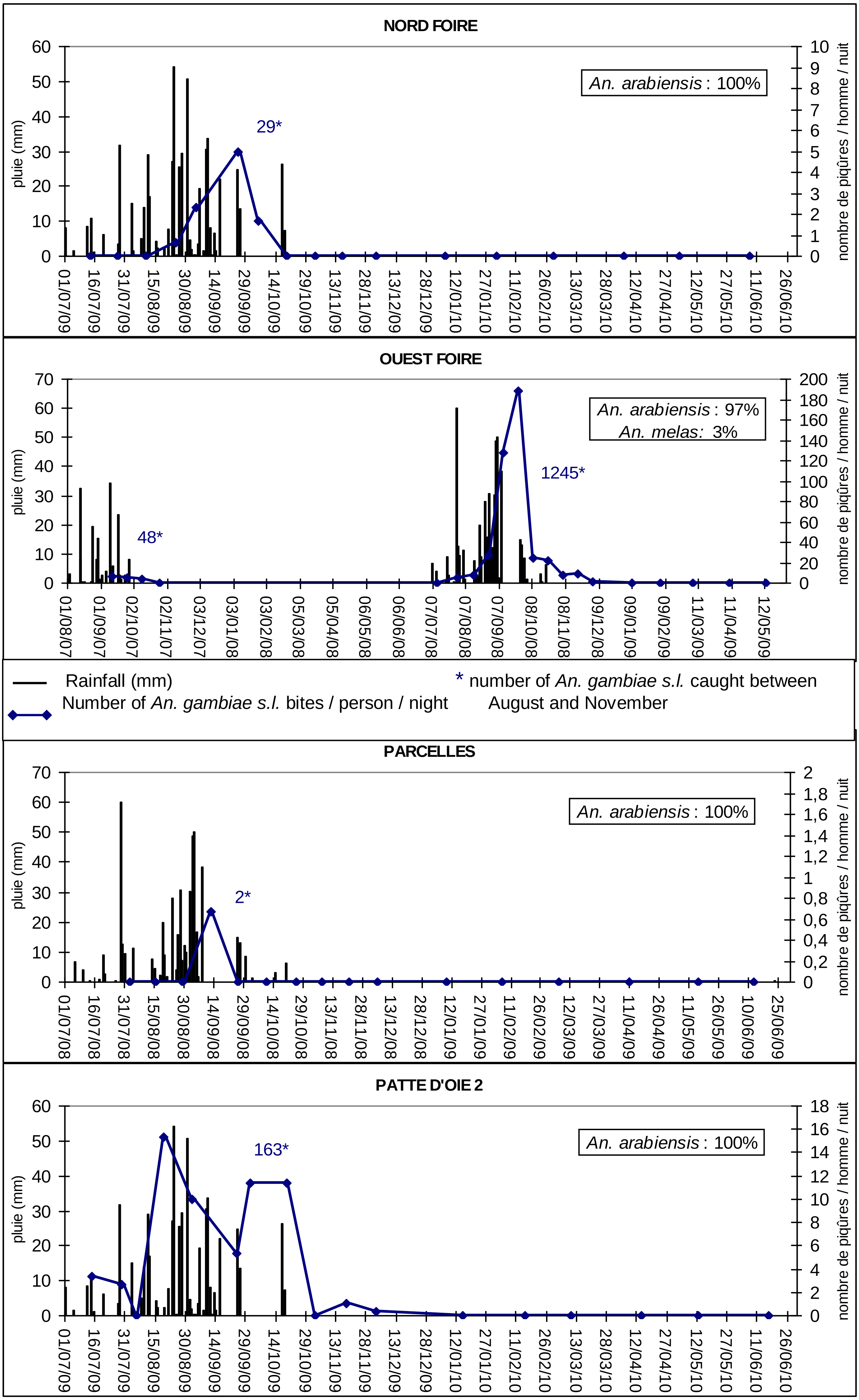
NORD FOIRE
An. arabiensis : 100%
29*
pluie (mm)
nombre de piqûres / homme / nuit
OUEST FOIRE
An. arabiensis : 97%
An. melas: 3%
48*
1245*
Rainfall (mm)
Number of An. gambiae s.l. bites / person / night
* number of An. gambiae s.l. caught between August and November
PARCELLES
An. arabiensis : 100%
2*
PATTE D'OIE 2
An. arabiensis : 100%
163*

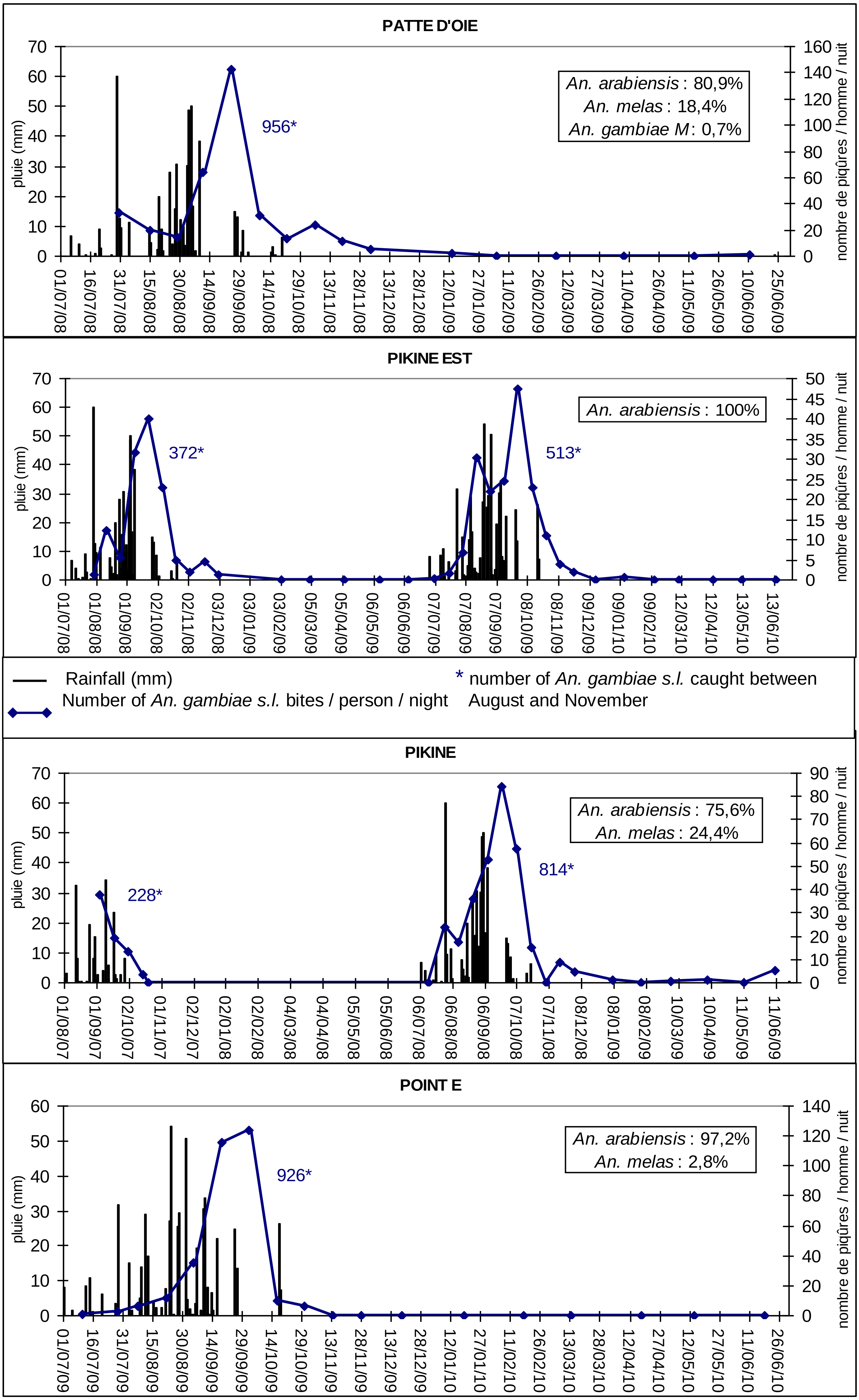
PATTE D'OIE
pluie (mm)
nombre de piqûres / homme / nuit
An. arabiensis : 80,9%
An. melas : 18,4%
An. gambiae M : 0,7%
956*
PIKINE EST
An. arabiensis : 100%
372*
513*
Rainfall (mm)
Number of An. gambiae s.l. bites / person / night
* number of An. gambiae s.l. caught between August and November
PIKINE
An. arabiensis : 75,6%
An. melas : 24,4%
228*
814*
POINT E
An. arabiensis : 97,2%
An. melas : 2,8%
926*

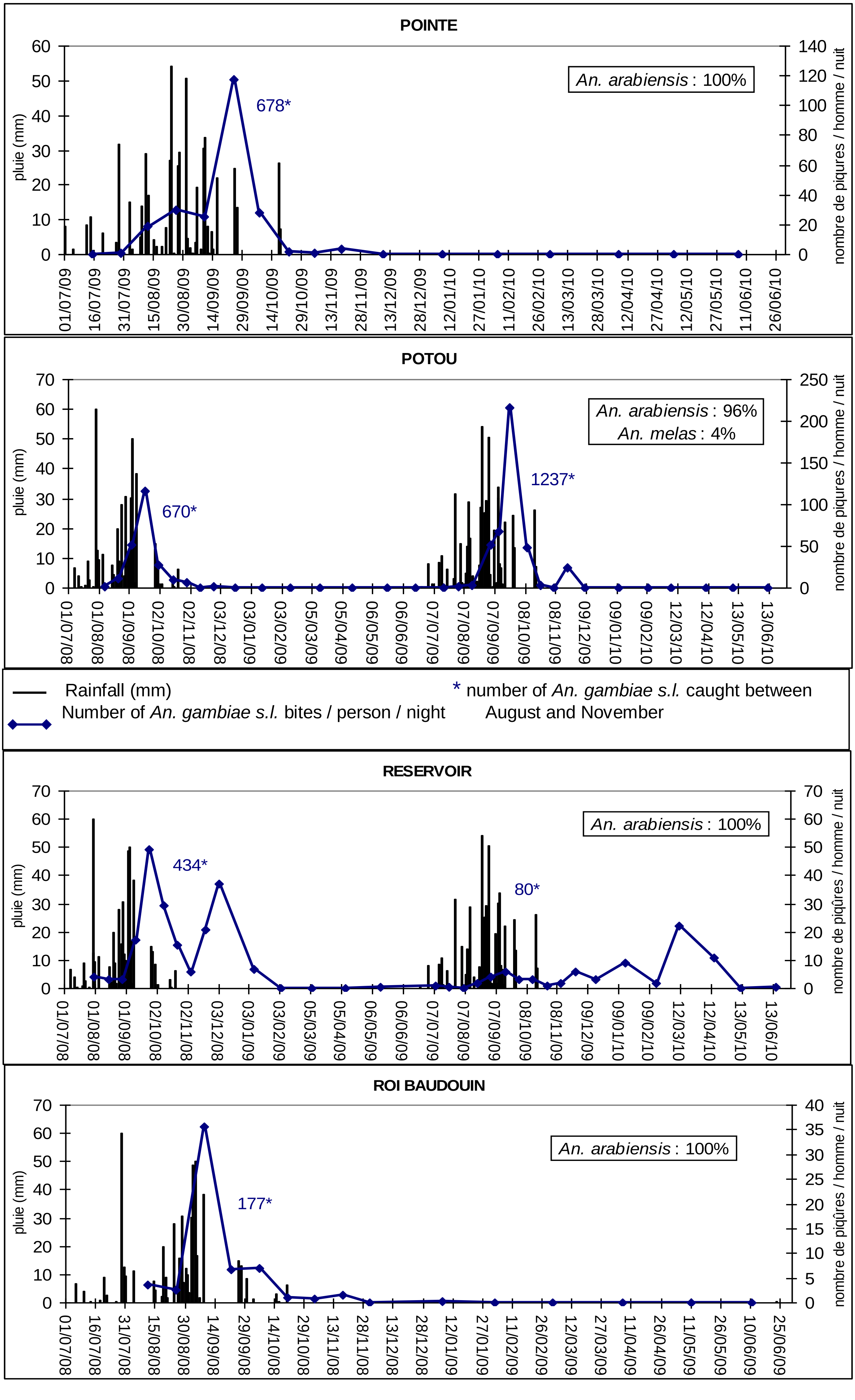
POINTE
An. arabiensis : 100%
678*
pluie (mm)
nombre de piqures / homme / nuit
POTOU
An. arabiensis : 96%
An. melas : 4%
670*
1237*
pluie (mm)
nombre de piqures / homme / nuit
Rainfall (mm)
Number of An. gambiae s.l. bites / person / night
* number of An. gambiae s.l. caught between August and November
RESERVOIR
An. arabiensis : 100%
434*
80*
pluie (mm)
nombre de piqûres / homme / nuit
ROI BAUDOUIN
An. arabiensis : 100%
177*
pluie (mm)
nombre de piqûres / homme / nuit

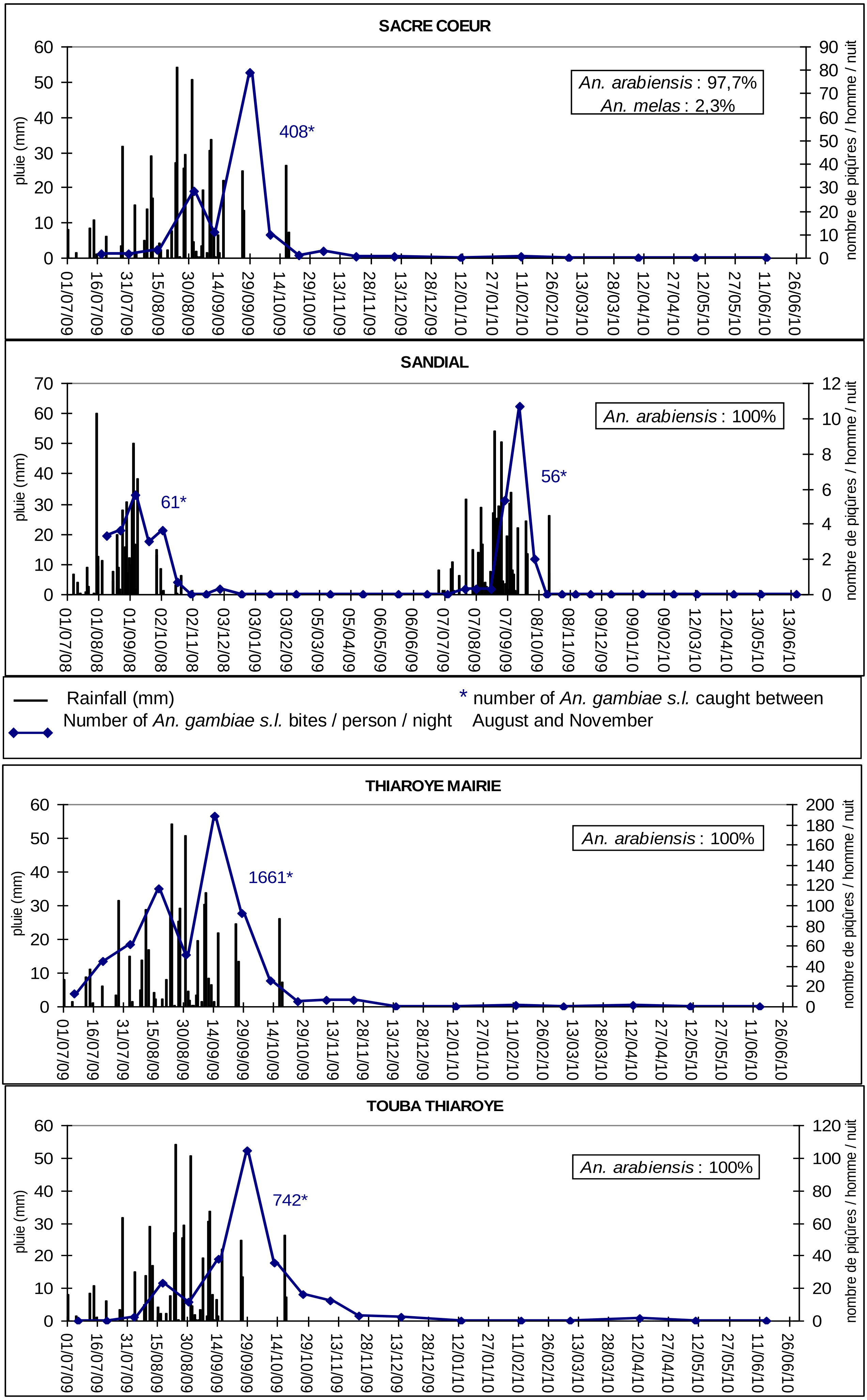
SACRE COEUR
pluie (mm)
nombre de piqûres / homme / nuit
An. arabiensis : 97,7%
An. melas : 2,3%
408*
SANDIAL
An. arabiensis : 100%
61*
56*
Rainfall (mm)
Number of An. gambiae s.l. bites / person / night
* number of An. gambiae s.l. caught between August and November
THIAROYE MAIRIE
An. arabiensis : 100%
1661*
TOUBA THIAROYE
An. arabiensis : 100%
742*

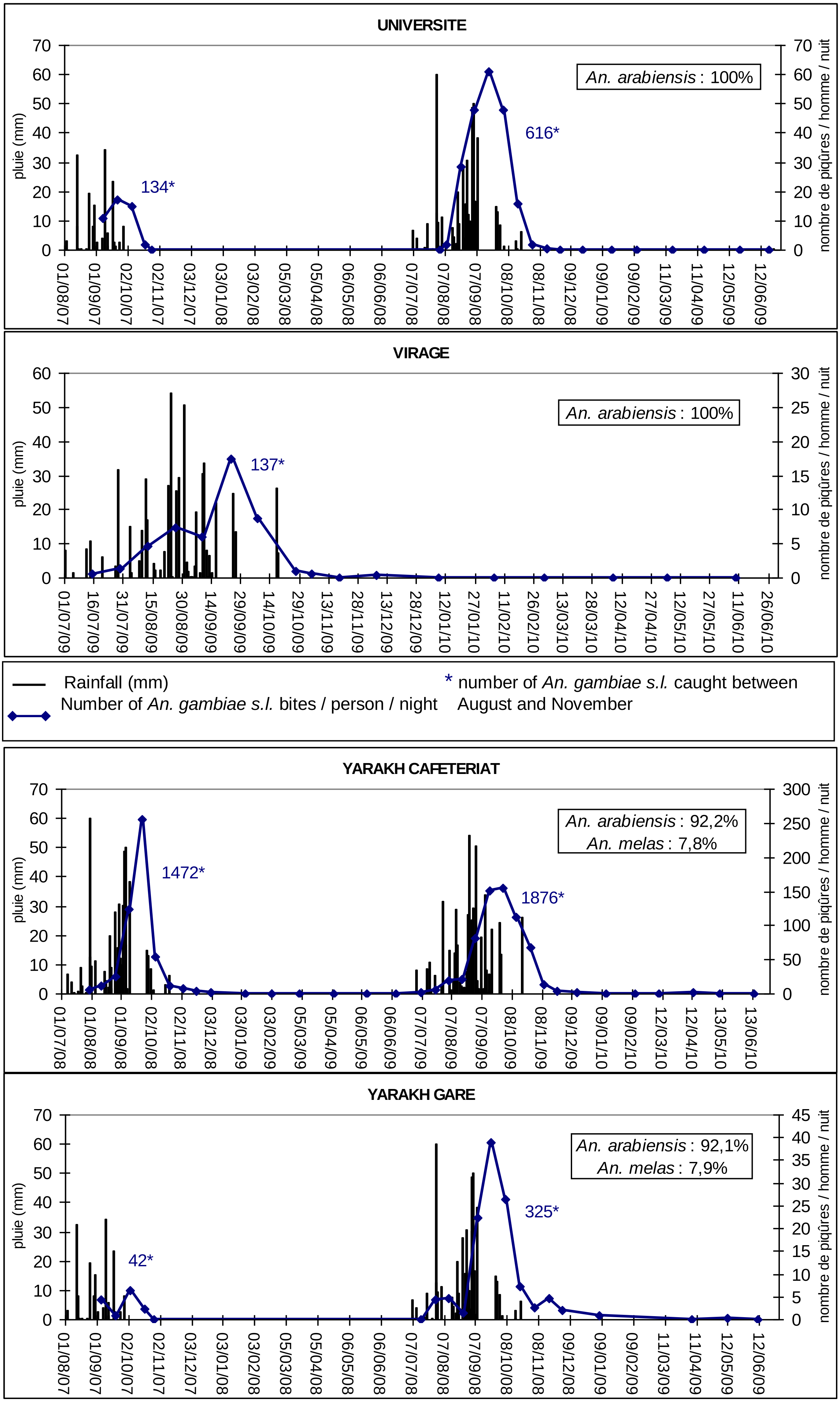
UNIVERSITE
pluie (mm)
nombre de piqûres / homme / nuit
An. arabiensis : 100%
134*
616*
VIRAGE
pluie (mm)
nombre de piqûres / homme / nuit
An. arabiensis : 100%
137*
Rainfall (mm)
Number of An. gambiae s.l. bites / person / night
* number of An. gambiae s.l. caught between August and November
YARAKH CAFETERIAT
pluie (mm)
nombre de piqûres / homme / nuit
An. arabiensis : 92,2%
An. melas : 7,8%
1472*
1876*
YARAKH GARE
pluie (mm)
nombre de piqûres / homme / nuit
An. arabiensis : 92,1%
An. melas : 7,9%
42*
325*

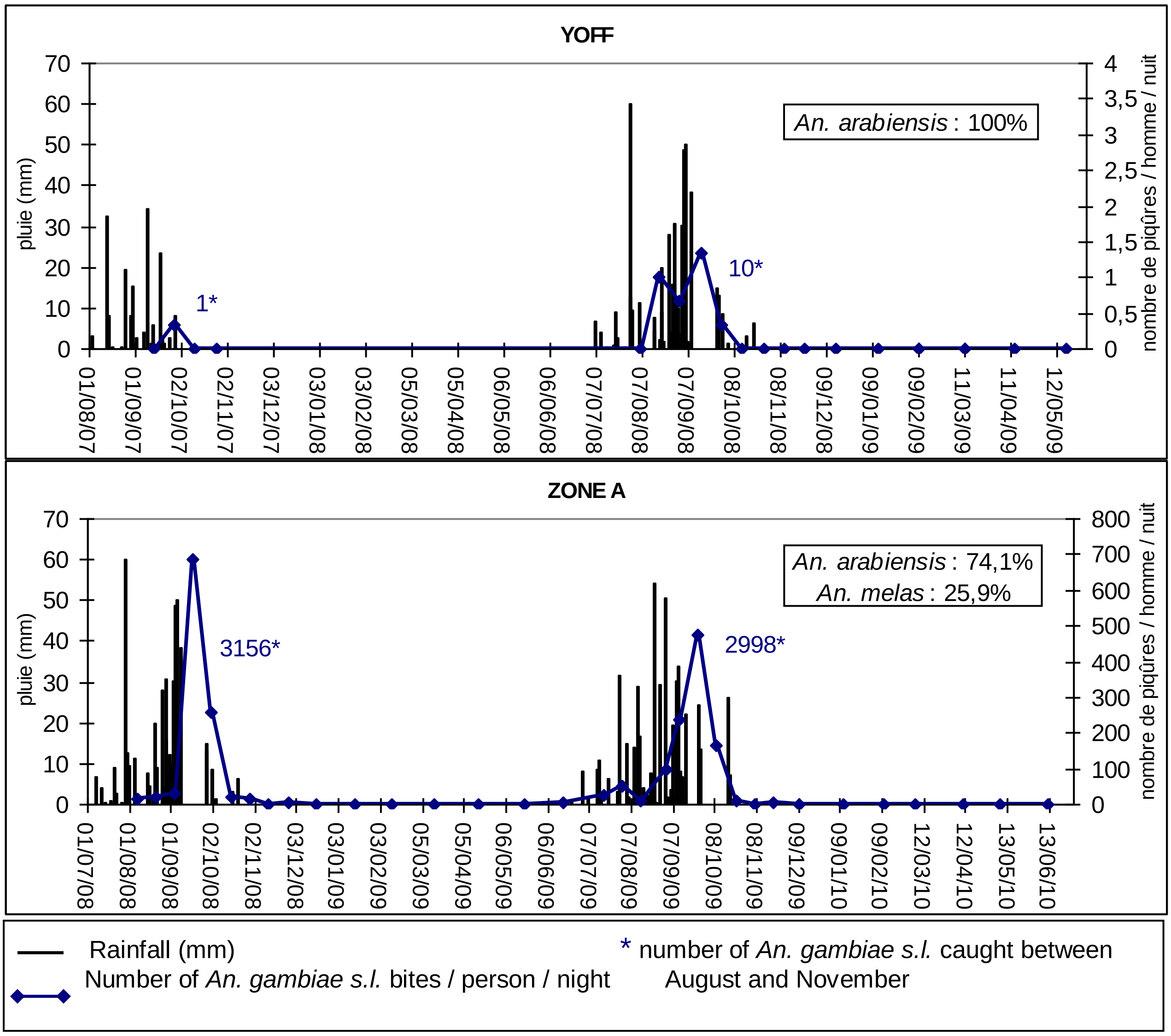
YOFF
pluie (mm)
nombre de piqûres / homme / nuit
70
60
50
40
30
20
10
0
4
3,5
3
2,5
2
1,5
1
0,5
0
An. arabiensis : 100%
1*
10*
01/08/07
01/09/07
02/10/07
02/11/07
03/12/07
03/01/08
03/02/08
05/03/08
05/04/08
06/05/08
06/06/08
07/07/08
07/08/08
07/09/08
08/10/08
08/11/08
09/12/08
09/01/09
09/02/09
11/03/09
11/04/09
12/05/09
ZONE A
pluie (mm)
nombre de piqûres / homme / nuit
70
60
50
40
30
20
10
0
800
700
600
500
400
300
200
100
0
An. arabiensis : 74,1%
An. melas : 25,9%
3156*
2998*
01/07/08
01/08/08
01/09/08
02/10/08
02/11/08
03/12/08
03/01/09
03/02/09
05/03/09
05/04/09
06/05/09
06/06/09
07/07/09
07/08/09
07/09/09
08/10/09
08/11/09
09/12/09
09/01/10
09/02/10
12/03/10
12/04/10
13/05/10
13/06/10
Rainfall (mm)
Number of An. gambiae s.l. bites / person / night
* number of An. gambiae s.l. caught between August and November
