# Supplementary material for: Conditions of malaria transmission in Dakar from 2007 to 2010
Source: Malar J. 2011 Oct 21;10:312. doi: 10.1186/1475-2875-10-312 (PMC3216462; doi:10.1186/1475-2875-10-312)
Supplement: Additional file 5 — Annual CSP index of each studied area, annual CSP index averaged for all studied areas and An. gambiae s.l. Rainy EIR (Sept-Oct) for each of the 45 studied areas in Dakar in Sept-Oct 2007 and Jul 2008-Jun 2010. [file 1475-2875-10-312-S5.PDF]

	Sept-Oct 2007 (10 areas)					Jul 2008-Jun 2009 (30 areas)					Jul 2009-Jun 2010 (30 areas)				
Studied areas	Rainy HBR	Annual CSP index for each area	Rainy EIR for each area	Annual CSP index averaged for all areas	Rainy EIR averaged for all areas	Rainy HBR	Annual CSP index for each area	Rainy EIR for each area	Annual CSP index averaged for all areas	Rainy EIR averaged for all areas	Rainy HBR	Annual CSP index for each area	Rainy EIR for each area	Annual CSP index averaged for all areas	Rainy EIR averaged for all areas
Almadies	43.7	0.76%	19.93	0.64%	16.78	160.8	0.09%	8.68	0.09%	8.68					
Pikine	19	0.44%	5.02	0.64%	7.3	52.3	0%	0	0.09%	2.82					
Université	11.5	0%	0	0.64%	4.42	43.1	0%	0	0.09%	2.33					
Hann (IRD)	5.3	0%	0	0.64%	2.04	28.8	0.37%	6.39	0.09%	1.56					
Ouest Foire	4.1	2.04%	5.02	0.64%	1.57	90.7	0.30%	16.33	0.09%	4.90					
Yarakh	3.5	2.38%	5	0.64%	1.34	23.8	0%	0	0.09%	1.29					
Gibraltar	3.6	0%	0	0.64%	1.38	18.5	0%	0	0.09%	1.00					
Liberté 5	0.7	0%	0	0.64%	0.27	2.5	0%	0	0.09%	0.14					
Grand Médine	0.3	0%	0	0.64%	0.12	0.3	0%	0	0.09%	0.02					
Yoff	0.1	0%	0	0.64%	0.04	0.6	0%	0	0.09%	0.03					
Patte d’Oie (1)						62.8	0.18%	6.78	0.09%	3.39					
Roi Baudoin						12.6	0%	0	0.09%	0.68					
Médina						0.7	0%	0	0.09%	0.04					
Grand Yoff						0.6	0%	0	0.09%	0.03					
Parcelles						0.2	0%	0	0.09%	0.01					
Cambérène						1.2	0%	0	0.09%	0.06	3.9	0%	0	0.12%	0.28
Bourguiba						1.8	0%	0	0.09%	0.10	0.8	0%	0	0.12%	0.06
Sandial						3.3	0%	0	0.09%	0.18	4.5	0%	0	0.12%	0.32
BA 160						9.6	0%	0	0.09%	0.52	17.3	0%	0	0.12%	1.25
Dial Diop						10.8	0%	0	0.09%	0.58	14.8	0%	0	0.12%	1.07
Karack						15.2	0.41%	3.74	0.09%	0.82	27.2	0%	0	0.12%	1.96
BIMA						17.6	0%	0	0.09%	0.95	74.1	0%	0	0.12%	5.34
Réservoir						24.8	0.48%	7.14	0.09%	1.34	4.1	0%	0	0.12%	0.30
Pikine Est						25.0	0.25%	3.75	0.09%	1.35	29.3	0.18%	3.16	0.12%	2.11
BA Ouakam						34.0	0.23%	4.69	0.09%	1.84	39.9	0.32%	7.66	0.12%	2.87
Potou						50.8	0%	0	0.09%	2.74	95.6	0.08%	4.59	0.12%	6.88
Fana						61.1	0%	0	0.09%	3.30	46.1	0.14%	3.87	0.12%	3.32

	Sept-Oct 2007 (10 areas)					Jul 2008-Jun 2009 (30 areas)					Jul 2009-Jun 2010 (30 areas)				
Studied areas	Rainy HBR	Annual CSP index for each area	Rainy EIR for each area	Annual CSP index averaged for all areas	Rainy EIR averaged for all areas	Rainy HBR	Annual CSP index for each area	Rainy EIR for each area	Annual CSP index averaged for all areas	Rainy EIR averaged for all areas	Rainy HBR	Annual CSP index for each area	Rainy EIR for each area	Annual CSP index averaged for all areas	Rainy EIR averaged for all areas
Cafetériat						114.5	0%	0	0.09%	6.18	124.4	0.26%	19.41	0.12%	8.96
Golf						171.6	0.03%	3.09	0.09%	9.27	115.8	0.21%	14.59	0.12%	8.34
Zone A						248.9	0.03%	4.48	0.09%	13.44	244.8	0.12%	17.63	0.12%	17.63
Castor											0	0%	0	0.12%	0.00
Nord Foire											2.3	0%	0	0.12%	0.17
Doro Aw											8	0%	0	0.12%	0.58
Virage											8.3	0%	0	0.12%	0.60
Patte d'Oie (2)											9.5	0%	0	0.12%	0.68
HLM											10.8	0%	0	0.12%	0.78
Liberté 6 Extension											17.3	0.43%	4.46	0.12%	1.25
Maristes											17.3	0%	0	0.12%	1.25
Sacré Coeur											32	0.45%	8.64	0.12%	2.30
Mamelles											37.3	0.20%	4.48	0.12%	2.69
Pointe des Almadies											43.3	0%	0	0.12%	3.12
Touba Thiaroye											47.4	0.12%	3.41	0.12%	3.41
Point E											71.1	0.10%	4.27	0.12%	5.12
Thiaroye Mairie											89.3	0.05%	2.68	0.12%	6.43
Dalifort											178.3	0.06%	6.42	0.12%	12.84
All areas	9.2			0.64%	3.5	42.9			0.09%	2.3	47.2			0.12%	3.4

Additional file 5. Annual CSP index of each studied area, annual CSP index averaged for all studied areas and *An. gambiae s.l.* Rainy EIR (Sept-Oct) for each of the 45 studied areas in Dakar in Sept-Oct 2007 and Jul 2008-Jun 2010
